# Effect of extrusion parameters and feed composition on physical characteristics, aroma profile and acrylamide content in pea protein-enriched corn extrudates^☆^

**DOI:** 10.1016/j.fochx.2026.104019

**Published:** 2026-05-22

**Authors:** Neslihan Göncüoğlu Taş, Dimitris P. Balagiannis, Sameer Khalil Ghawi, Vural Gökmen, Jane K. Parker

**Affiliations:** a Food Quality and Safety (FoQuS) Research Group, Department of Food Engineering, Hacettepe University, 06800 Beytepe, Ankara, Türkiye; b Department of Food and Nutritional Sciences, University of Reading, Whiteknights, Reading RG6 6DZ, UK

**Keywords:** Twin-screw extruder, Pea protein isolate, Maillard reaction, Aroma, Acrylamide, Snack

## Abstract

Low-moisture extrusion is gaining relevance for high-protein snacks, yet its effect on product quality and safety remain insufficiently understood. In this study, the effects of pea protein isolate content (0–70%), moisture (15–17%), and extrusion parameters, including screw speed (400–500 rpm) and barrel temperature (up to 145–160 °C) were studied using a twin-screw extruder. Product characteristics were evaluated in terms of bulk density, dimensions, texture, colour, and aroma profile while acrylamide levels were quantified to assess safety. Feed composition had a greater influence than extrusion parameters. Among the investigated processing conditions, formulations containing up to 50% pea protein represented a practical optimum, providing relatively better physical properties and enhanced aroma formation while maintaining low acrylamide levels, whereas higher protein incorporation (70%) led to pronounced and less desirable changes. Overall, low-moisture extrusion is promising for high-protein snacks with low acrylamide risk.

## Highlights

• Feed protein level is more critical than extrusion parameters on extrudate properties.
• Screw speed, temperature, moisture did not affect physical properties of extrudates.
• Acrylamide was below benchmark level in corn-based high protein extrudates.
• Pea protein isolate addition (50%) to corn-based feed promoted key pyrazines.
• Increasing temperature promoted pyrazines formation but slower screw speed did not.

## 1 Introduction

Increased consumer awareness has created a need for more plant-based high-protein foods in the market. Pea (*Pisum sativum* L.), a member of legume family, is rich in carbohydrates (40–50% of starch), protein (20–25%), dietary fibre (10–20%), vitamins, and minerals (Lu et al., 2020; Tulbek et al., 2024). Pea protein is obtained from peas through dry or wet processing. Dry processing yields protein concentrate (< 80%), while wet processing produces protein isolate (> 80%) (Liu et al., 2023; Sridharan et al., 2020). Pea protein is considered as an important ingredient because of its availability, affordability, low allergenicity, and being obtained from an environmentally sustainable crop (Shanthakumar et al., 2022). It not only enhances the protein content of food products but also influences their texture and structure through its solubility, viscosity, water- and oil-binding properties, as well as its emulsifying, foaming, and gelation capacities (Ge et al., 2020; Liu et al., 2023; Shanthakumar et al., 2022). Accordingly, it is widely utilised in the food industry as an emulsifier, encapsulating agent, biodegradable natural polymer, and functional ingredient that improves protein content and/or contributes to textural attributes of cereal, bakery, dairy, and meat products (Shanthakumar et al., 2022). The pea protein isolate market size grew rapidly from US$ 1.24 billion in 2025 to US$ 1.44 billion in 2026, reflecting a compound annual growth rate of 15.7%, and is expected to reach US$ 2.56 billion by 2030 (The Business Research Company, 2026). The global extruded snack market size was valued at US$ 62.43 billion in 2025 and projected to grow US$ 65.90 billion in 2026 and to US$ 103.1 billion by 2034. Importantly, corn-based extruded snacks hold a major market share of 46.69% in 2026 (Fortune Business Insights, 2026). According to recent market analyses, consumers are seeking protein- and fibre-rich extruded products, creating substantial opportunities for manufacturers to develop innovative formulations with novel flavours, textures, and shapes (Fortune Business Insights, 2026).

Extrusion is a process where both thermal and mechanical energy is applied to the materials while they are mixing with water. Molten material moves by the effect of screw rotation in the barrel under high pressure, shear, and changing temperature conditions. The material then passes through a die, where the rapid evaporation of water generates puffed structure. Processing parameters, including barrel temperature profile, screw speed, feed moisture, and feed rate, play critical roles in determining the final characteristics of the extruded product (Mosibo et al., 2022). Extruded snacks are commonly produced from starch-based ingredients such as corn, rice, or wheat, since starch gelatinization plays a key role in creating the desirable expanded and porous structure. Typical low-moisture extrusion is carried out at moisture contents below 35% (Guo et al., 2024), most commonly in the range of 12–24%, with processing temperatures reaching up to 180 °C and screw speeds between 50 and 550 rpm, depending on the material used (Pasqualone et al., 2020).However, efforts to increase the protein content of these products remain challenging, as higher protein levels often interfere with proper structure formation. Importantly, protein addition affects both the physical attributes and the flavour profile of the final product, and these effects vary according to the protein source and processing conditions.
Aroma notes originating from pea protein isolate have been described as beany and green, mostly originating from pea itself or lipid oxidation products, which makes pea protein challenging to incorporate into recipes (Xiang et al., 2023). Because extrusion combines mechanical shear and thermal treatment, the stability of aroma-active compounds can be substantially influenced by the processing conditions, potentially leading to the reduction or removal of undesirable aroma compounds (Li et al., 2024a). Additionally, extrusion may promote the formation of desirable aroma compounds and the reduction of unwanted ones, largely through molecular interactions that favour Maillard reaction (Ma et al., 2026). However, Maillard reaction can also result in the formation of acrylamide in extruded products (Masatcioglu et al., 2014). Acrylamide has been classified by the International Agency for Research on Cancer (IARC) as a Group 2 A “probably carcinogenic to humans” compound (International Agency for Research on Cancer (IARC), 1994). It is formed during the thermal processing of foods, especially at high temperatures (*T* > 120 °C) and prolonged processing times, through reactions between asparagine and reducing sugars under low-moisture conditions (European Food Safety Authority (EFSA), 2015; Mottram et al., 2002; Stadler et al., 2002). Although the processing time is shorter than that of other thermal treatments such as baking or roasting, extrusion can reach similarly high temperatures (Berk, 2009). Consequently, it is essential to elucidate the effects of the extrusion process on the concurrent formation and/or reduction of aroma compounds and acrylamide.
It is hypothesized that extrusion processing, depending on the extrusion parameters, enables the reduction of undesirable aromas and the formation of novel desirable flavour compounds, facilitating protein-enriched snack production, while increased protein content may influence physical properties and acrylamide formation. Hence, the present study investigated the effects of feed composition (pea protein isolate and moisture content) and extrusion parameters (screw speed and barrel temperature) on the chemical (acrylamide and aroma) and physical properties (bulk density, hardness, fracturability, and colour) of extruded cornmeal-pea protein snacks.

## 2 Materials and methods

### 2.1 Chemicals and consumables

Pea protein isolate (80% protein) was purchased from Nutraceuticals Group Europe (Merstham, UK) and cornmeal medium (67% carbohydrate, 13% fat, 7% protein, 0.4% fibre) from Natco Foods Limited (Buckingham, UK). 2-Isopropylpyrazine (99.5%) was obtained from Alfa Aesar (Waltham, MA, USA). 3-Methyl-2-butanone (99%), 3-furaldehyde (97 + %), and the alkane standards C_5_-C_22_ (100 μg/mL in diethyl ether) and all the other aroma chemicals were obtained from Sigma-Aldrich (Gillingham, UK). Acrylamide (≥ 99%), potassium hexacyanoferrate (Carrez I), zinc sulfate heptahydrate (Carrez II), formic acid (LC-MS grade), and methanol (LC-MS grade) were purchased from Merck (Darmstadt, Germany). Acrylamide-d_3_ was obtained from Toronto Research Chemicals (Manitoba, ON, Canada). Deionized water (0.05 μS/cm, Barnstead Smart2Pure, Thermo Fisher Scientific, Waltham, MA, USA) was used during all analyses.

### 2.2 Preparation of cornmeal-pea protein isolate dry feed

A preconditioning step was applied to the solid feed to agglomerate the granules of cornmeal-pea protein isolate mixture, prevent component separation and improve flowability during gravimetric feeding. For preconditioning, 1 kg of cornmeal: pea protein isolate mixtures were prepared at ratios of 100:0, 85:15, 70:30, 50:50, and 30:70 (*w/w*). Each mixture was first dry-mixed manually using a whisk and subsequently transferred to a mixer (Kenwood, UK), where 300 mL of drinking water was added and mixed thoroughly. The wet mixtures were then passed through a sieve to obtain a uniform particle size for feeding. Thereafter, the mixtures were dried in a tray oven at 70 °C for 24 h.

### 2.3 Extrusion of pea protein enriched corn extrudates

A twin-screw extruder (Process 16 Hygienic, Thermo Fisher Scientific, Germany) was used to produce cornmeal-pea protein isolate extrudate equipped with a 3 mm diameter die head. Screws were placed in a barrel with eight temperature-controlled heating zones with the screw length/diameter ratio of 40. Pressure was measured with a pressure probe on the exit zone of the barrel. The dry feed was introduced into the first zone using a gravimetric feeder, while the liquid feed was introduced into the second zone using a peristaltic pump. Temperature profile (40–60–80–100–120–140–145–145 °C), screw speed (400 rpm), and dry feed rate (3.4 kg/h) remained constant, while dry feed composition (cornmeal-pea protein ratio of 100:0, 85:15, 70:30, 50:50, and 30:70, *w/w*), moisture content (15, 16, and 17%), and the corresponding liquid feed rate were varied to assess the effect of composition on physical and chemical properties of extrudates. Preliminary extrusion trials were conducted to determine operating conditions that avoided process instability such as torque fluctuations, die blockage, or unstable extrusion flow.

Effect of changing in screw speed (400, 450, and 500 rpm) and temperature profile on the physical and chemical properties of extrudates were evaluated by using 50:50 and 30:70 cornmeal-pea protein isolate mixtures as dry feed, with a moisture content of 15%. These formulations were selected because they more clearly reflect protein-driven effects on processing, as opposed to starch-dominant behaviour observed at lower pea protein isolate levels. Pressure (bar), torque (Nm), melt temperature (°C) which is the actual temperature of the material inside the barrel, and specific mechanical energy (kWh/g) which represents the mechanical work imparted per unit mass of material, were recorded for each extrusion. Bulk density (g/mL) and moisture content of the dry feed were measured to calculate the overall liquid feed rate (mL/min), which was adjusted to achieve the desired initial feed moisture content.

### 2.4 Chemical analysis of cornmeal and pea protein isolate

#### 2.4.1 Determination of sugars by high-performance anion exchange liquid chromatography coupled amperometric detector

Sugars were extracted from 0.25 g of pea protein isolate and cornmeal using 5 mL of water in the presence of 125 μL each of Carrez I and Carrez II solutions. Samples were vortex-mixed to ensure thorough dispersion, followed by centrifugation at 9500 ×*g* for 5 min. The resulting supernatants were filtered through a 0.2 μm nylon filter and collected in vials for subsequent analysis. Analysis of sugars was performed according to the method described by Stewart et al. (2018) with some modifications. A high-performance anion exchange liquid chromatography coupled to an amperometric detector (HPAEC-PAD) was used for the analysis. A Thermo Dionex ICS - 6000 system (Dionex Corp., Sunnyvale, CA) consisting of an ICS 6000 gradient pump, an electrochemical detector with a gold working electrode, an ICS 6000 SP oven, and an AS-AP autosampler with tray cooling option was operated for the injections. The reference electrode was PdH and the waveform was gold-carbo-quad. The detector was in IntAmp mode. Separation was performed using a pellicular anion-exchange resin-based column, CarboPac PA-1 analytical (4 mm × 250 mm), connected to a CarboPac PA1 Guard (4 mm × 50 mm) (Dionex Corp., Surrey, UK). The column was maintained at 20 °C; elution was performed at a flow rate of 1 mL/min using gradient concentrations of sodium hydroxide and water solutions. There was a multi-step gradient programme where A and D were water, B was 16 mM NaOH solution, C was 250 mM NaOH solution. The gradient started with 35% A, 10% C and 55% D for 25 min, then NaOH concentration increased by setting the pump 35% A, 50% C, 15% D for 5 min, the settings were constant for 10 min, returned to initial conditions within 5 min and kept constant for 5 min at the initial conditions. The total run time was 70 min. The injection volume was 20 μL. All chromatographic analyses were performed in triplicate. Standard solutions of glucose and fructose (0.1–2 mg/L), sucrose (1–10 mg/L), and maltose (1–10 mg/L) were analysed under the same conditions for the calibration curve. Chromatographic analysis of sugar peaks was performed on Chromeleon Software (Dionex Corp., Sunnyvale, CA).

#### 2.4.2 Determination of free amino acids by high-performance liquid chromatography coupled to triple quadrupole mass spectrometer

Free amino acids were extracted from 1.0 ± 0.05 g of pea protein isolate and cornmeal through three consecutive extractions with 10, 5, and 5 mL of water. After each extraction, samples were vortexed for 5 min and centrifuged at 6654 ×*g* for 5 min. The resulting supernatants were combined, and 0.2 mL of the pooled extract was mixed with 0.8 mL acetonitrile, centrifuged, and filtered through a 0.2 μm PTFE membrane (Fisher Scientific, Leicestershire, UK) into an autosampler vial. External calibration curves of amino acids were built from 1 to 50 μM in water:acetonitrile mixture (20,80, *v,v*). Amino acids were analysed using an Agilent 1200 high-performance liquid chromatography (HPLC) system coupled to a 6410 triple quadrupole mass spectrometer with electrospray ion source (ESI) in positive mode (Agilent Technologies, Santa Clara, CA, USA). The chromatographic conditions and identification of amino acids in multiple reaction monitoring mode (MRM) using the ion transitions were as previously reported (Kocadaǧli et al., 2013).

### 2.5 Determination of volatile compounds by solid phase micro extraction gas chromatography mass spectrometry

Analysis of volatile compounds of cornmeal, pea protein isolate, and extruded snacks was performed as described by Göncüoğlu Taş et al. (2023) with some modifications. A 1 g portion of ground extruded sample was transferred into 20 mL screw cap glass headspace vials. To equalise the ionic strength of the model systems and enhance the release of the volatile compounds into the headspace, 2 mL of saturated NaCl solution (35% NaCl) containing a mixture of 3-methyl-2-butanone and 3-furaldehyde (0.5 mg/L), and isopropylpyrazine (0.05 mg/L) as internal standards was added into vials. A CTC PAL headspace autosampler (CTC Analytics AG, Switzerland) was used for solid-phase microextraction (SPME). The autosampler was coupled to an Agilent 7890 A gas chromatography (GC) system (Agilent Technologies, Santa Clara, CA, USA) attached to a 5975C inert MSD triple-axis detector. A 50/30 μm DVB/CAR/PDMS SPME fibre (Supelco, Bellefonte, PA, USA) was used for the extraction of the volatile compounds. Agitation for 30 min at 60 °C and extraction for 20 min at 60 °C was applied to the samples. The fibre was desorbed in the injection port at 250 °C. Helium was the carrier gas and its flow rate was constant (1.2 mL/min). A DB-5MS column (30 m × 0.25 mm × 0.25 μm film thickness, Agilent, Santa Clara, CA, USA) was employed for the separation of the volatile compounds. GC oven temperature was held at 40 °C for 2 min initially. Then, it was raised to 280 °C at a rate of 4 °C/min. Mass spectrometric conditions and the identification and semi-quantification of volatile compounds were conducted according to the method described by Göncüoğlu Taş et al. (2023). Retention times of the compounds, calculated LRI, authentic LRI, and the ions selected for semi-quantification are presented in **Supplementary Material, Table S1**. The area of 3-furaldehyde for furans, isopropylpyrazine for pyrazines, 3-methyl-2-butanone for Strecker aldehydes and the rest of the compounds were used for calculations.

### 2.6 Determination of acrylamide by ultra performance liquid chromatography coupled to tandem mass spectrometry

A multiple-stage extraction technique was performed as described before (Gökmen et al., 2009). Ground sample (100 mg) was extracted with 10 mM formic acid in water containing 10 μg/L acrylamide-d_3_ in triple-stage by using 2 mL extraction solution in total. The first step of the extraction involved a Carrez clarification step. The combined extract was cleaned up by passing through an Oasis MCX cartridge (Waters, Milford, MA, USA). Acrylamide was analysed by a Waters Xevo TQ-S cronos Triple Quadrupole MS coupled with a Waters Acquity H Class Plus UPLC (Waters Corporation, Milford, MA, USA) under the chromatographic conditions and ESI source parameters described previously (Göncüoğlu Taş et al., 2023). The MRM transitions for acrylamide-d_3_ was *m*/*z* 75 to 58 (fragmentor voltage of 70 V, collision energy of 9 V). The first quadrupole was set to unit resolution, and the second quadrupole was set to wide resolution for each MRM transition. Acrylamide was quantified by a linear calibration curve built in 0.1 and 10 μg/L range (containing 10 μg/L acrylamide-d_3_). The limit of detection (LOD) and limit of quantification (LOQ), based on signal-to-noise ratios of 3 and 10, were 0.08 and 0.26 μg/kg, respectively, for acrylamide.

### 2.7 Moisture analysis

Moisture content of the feed and extrudates was analysed by using an infrared moisture analyzer (Sartorius MA150, Göttingen, Germany) at 105 °C. All measurements were carried out in triplicate.

### 2.8 Bulk density analysis

The bulk density of the feed was determined as described by Chandra et al. (2015) with some modifications. The tared 250 mL volumetric cylinder was filled with the feed, and its weight was recorded. Bulk density (g/mL) was calculated as the ratio of feed weight to 250 mL. Measurements were performed in triplicate. (Chandra et al., 2015).

Bulk density of the extrudates was determined by volumetric displacement method described by Meng et al. (2010) with minor modifications. The bottom 10 mL of the 250 mL volumetric cylinder was filled with glass beads after which approximately 5 g of extrudates cut into approximately 2 cm pieces were placed into the cylinder. The remaining volume of the cylinder was then filled with glass beads of known bulk density. After tapping the cylinder ten times to ensure proper packing, glass beads were added again if necessary, and the surface was levelled before weighing. Measurements were performed in five replicates.

### 2.9 Determination of diameter

The diameter of the extrudates was measured using a digital vernier calliper. Ten different pieces of extrudates were used for measurements (Meng et al., 2010).

### 2.10 Texture analysis

Texture profile analysis of extruded snacks was performed using a TA.XT2 Texture Analyzer (Texture Technologies Corp., Scarsdale, NY/Stable Microsystems, Godalming, UK) by using three point bending test. The test mode was compression, test speed was 1 mm/s, target mode was distance, and the distance was 3 mm. Hardness (N) and fracturability (mm) were determined using ten pieces of extrudates at the same length.

### 2.11 Colour analysis

Colour (L*, a*, and b*) values of extrudates were measured by using a hand-held Minolta Chroma Meter (Minolta Camera Co., Osaka, Japan). Measurements were performed on ten individual pieces of extrudate sample. Chroma (C*) and hue angle (*h*°) values were calculated according to Eq. (1) and Eq. (2), respectively. Hue angle (*h*°) values were converted to a 0–360° scale by adding 360° whenever the calculated values were negative.

(1) $$C^{*}=\sqrt{a^{{*}^{2}}+{b^{*}}^{2}}$$

(2) $$h^{{}^{\circ}}=\tan^{-1}\left(\;\frac{b^{*}}{a^{*}}\right)$$

### 2.12 Statistical analysis

Data were analysed using analysis of variance (ANOVA), followed by Tukey's *post-hoc* test at *p* = 0.05, assuming that the data were normally distributed. The differences in the amino acid and sugar profile of raw material were determined by Student's *t*-test (*p* ≤ 0.05) using Microsoft Excel 2025 (Microsoft Corp., Washington, USA). The effects of feed composition and processing parameters on the formation of volatile compounds were summarized by principal component analysis (PCA) using XL - Stat (Addinsoft, Paris, France).

## 3 Results and discussion

### 3.1 Effect of feed composition and extrusion conditions on the physical characteristics of cornmeal-pea protein isolate extrudates

The processing parameters measured during extrusion and the material characteristics of the cornmeal (CM) - pea protein isolate (PPI) feed at varying moisture levels and compositions are summarized in Table 1. Five feed formulations (E1, E2, E3, E6, and E9) were prepared with pea protein isolate contents of 0, 15, 30, 50, and 70%, respectively, at a constant feed moisture content of 15%. Formulations E5 and E8 contained a CM:PPI ratio of 50:50 and 30:70, with a feed moisture content of 16%. Additionally, formulations E4 and E7 corresponded to feeds with a 50:50 and 30:70 CM:PPI and a moisture content of 17%, respectively. The barrel temperature profile (40–60–80–100–120–140–145–145 °C), the feed rate (3.4 kg/h) and the screw speed (400 rpm) were kept constant across all experiments. The SME values ranged from 540 to 684 kJ/kg while torque varied between 13.0 and 18.0 N.m depending on the PPI content of the feed at constant moisture (15%). Increasing the PPI content in the dry feed resulted in lower torque during extrusion, accompanied by a decrease in specific mechanical energy (SME). This can be explained by the decrease of the starch content of the feed, because starch is responsible from the increase in resistance to flow. The underlying mechanism of starch behaviour was reported by Rincón-Aguirre et al. (2025). Starch partially gelatinises, and the degree of order and double helices decreases, leading to reduced crystallinity of the material. This disorganised structure of starch exposed OH- groups, increasing the interaction between starch molecules and water, resulting in increased viscosity, higher consistency, greater elastic response, and a more cohesive matrix (Rincón-Aguirre et al., 2025). Supporting this mechanism, Dey et al. (2024) reported that sugar act as a plasticiser, restricting starch transformation and leading to decreased SME values during low-moisture extrusion of corn starch (Dey et al., 2024). Similarly, Muñoz et al. (2025) found lower SME values for hydrolysed pea protein compared with hydrolysed rice protein, which was attributed to differences in viscosity and hydration capacity that reduced material resistance during extrusion (Muñoz et al., 2025). A decrease in both torque and SME with increasing proportions of either chickpea flour (50 and 75%) or yellow pea concentrate (10, 20 and 40%) at constant in-barrel moisture content during low-moisture twin-screw extrusion of cornmeal was reported by Wang et al. (2022). In accordance, Usman et al. (2026) demonstrated that incorporating intermediate wheatgrass, a protein and fibre source, with pea starch resulted in SME values ranging from 586.40 to 1132.09 kJ/kg, with increasing pea starch content leading to higher SME values (Usman et al., 2026). Both the SME values and the effect of higher protein incorporation to the feed were consistent with the results of the present study.

**Table 1:** Process and material characteristics during extrusion of cornmeal-pea protein isolate feed compositions at varying moisture contents⁎, #.

	E1	E2	E3	E4	E5	E6	E7	E8	E9
	CM:PPI (100:0)-M15	CM:PPI (85:15)-M15	CM:PPI (70:30)-M15	CM:PPI (50:50)-M17	CM:PPI (50:50)-M16	CM:PPI (50:50)-M15	CM:PPI (30:70)-M17	CM:PPI (30:70)-M16	CM:PPI (30:70)-M15
Bulk Density of Dry Feed (g/mL)	0.74 ± 0.001	0.68 ± 0.002	0.58 ± 0.005	0.49 ± 0.003	0.49 ± 0.003	0.49 ± 0.003	0.49 ± 0.005	0.49 ± 0.005	0.49 ± 0.005
Moisture Content of Dry Feed (%)	10	10	10	3.3	3.3	3.3	3.45	3.45	3.45
Moisture Content of Feed (%)	15	15	15	17	16	15	17	16	15
Moisture Content of Extrudate (%)	10.3 ± 0.6	9.7 ± 0.4	9.4 ± 0.4	9.7 ± 0.7	9.5 ± 0.0	9.9 ± 0.8	9.7 ± 0.5	9.4 ± 0.3	9.7 ± 0.4
Flow Rate of Liquid Feed (mL/min)	3	3	3	9	8.5	8	9	8.5	8
Pressure (kPa)	3500	4000	5500	4800	4800	4800	3000	3500	4200
Torque (Nm)	18	18	16.5	13	14	14.4	11.8	13.3	13.3
Melt Temperature (°C)	150	150	154	148	151	152	147	147	147
Specific Mechanical Energy (kJ/kg)	684	684	684	612	576	540	504	540	540

⁎ Barrel temperature profile (°C): 40–60–80-100-120-140-145-145, screw speed (rpm): 400, and feed rate of dry feed (kg/h): 3.4 were same for each extrusion.

# E: extrudate, CM: cornmeal, PPI: pea protein isolate, M: moisture content.

Only a slight reduction in torque was observed when feed moisture increased from 15% to 17%, although maximum output remained unchanged. The decrease in torque can be attributed to the reduced friction within the barrel at higher moisture levels. Although a decrease in SME would be expected with decreasing torque, the SME values did not exhibit a consistent trend with moisture content, likely because the changes in moisture were too small to produce a noticeable effect. Singh et al. (2024) reported that increasing moisture content of the feed from 30 to 40% decreased die pressure, torque and SME during production of texturized vegetable proteins from lentil. Reduction in SME by increased water was explained by the lubricating effect of water leading reduction in feed viscosity and shear force (Kaur et al., 2022). Since it is difficult to provide an expansion to the extrudates at higher moisture levels and extruder pressure increases excessively at lower moisture contents, the feed was maintained within this narrow moisture range in this study. The same limitations about the range of feed moisture were also reported by Usman et al. (2026) that feed moisture below 19% created high torque and excessive pressure in the extruder leading to machine to stop, whereas moisture above 23% in the feed limited expansion during the extrusion of intermediate wheatgrass-pea starch blends.

Bulk density of the extrudates did not change significantly (*p >* 0.05) until pea protein isolate content in the feed was 70%, increasing from 0.16 g/mL in the cornmeal-only extrudate to 1.32 g/mL at 70% pea protein isolate (Table 2). Extrudate diameter decreased with increasing pea protein isolate addition to the feed, particularly at 70%, where it was reduced to one-third of that of cornmeal-only extrudate. The decrease in diameter and increase in bulk density of the extrudates with protein addition can be attributed to competition between protein and starch for water, resulting in reduced starch swelling or reduced molecular interactions among proteins. Similar behaviour has been also reported when quinoa and soybean added as a protein source in corn-based extruded snack formulations (Fatima et al., 2025). Korkerd et al. (2016) reported that partial substitution of corn grit with defatted soybean meal, germinated brown rice meal, and mango peel powder increased the protein and dietary fibre content, but decreased the expansion of corn-based extruded snacks. The hardness of extrudates showed only minor differences with pea protein isolate levels up to 50% in the feed, whereas a pronounced decrease was observed at 70%. Additionally, extrudates became more fracturable when pea protein isolate content in the feed reached 50% or higher. Protein addition to the feed, independent of the type of protein, was also reported to increase hardness of the product in extruded products (Hood-Niefer & Tyler, 2010). The increase in hardness with rising protein content in wheat - pulse extrudates has been attributed to heat- and shear-induced protein aggregation, involving the unfolding of native proteins and their subsequent crosslinking with components such as starch and lipids, forming a dense continuous matrix (Martin et al., 2020). The decrease in hardness of extrudates in the present study when 70% pea protein isolate incorporated into feed may be attributed to the dilution of the starch fraction in the feed matrix, which limits the extent of effective crosslinking or homogenous continuous matrix. Microstructural analysis might further explain the observed reduction in hardness by revealing alterations in surface properties, protein-starch network continuity, and pore wall thickness, void fraction of the extrudates (Philipp et al., 2017) and can be considered in the future studies. Moisture content of feed did not significantly (*p* > 0.05) influence bulk density, diameter, or hardness. The moisture content investigated in this study was confined to a narrow range due to processing constraints. The higher moisture levels limited extrudate expansion, whereas lower moisture contents resulted in excessive extruder pressures. As a result, the findings may not be directly extrapolated to extrusion conditions beyond this range.

**Table 2:** Physical properties of cornmeal-pea protein isolate extrudates with different compositions at varying moisture contents⁎, #.

		Bulk Density (g/mL)	Diameter (mm)	Hardness (N)	Fracturability (mm)	*L*⁎	*a*⁎	*b*⁎
E1	CM:PPI (100:0)-M15	0.16 ± 0.001^c#^	10.3 + 1.0^b^	7.0 ± 1.9^c^	28 ± 5^bc^	52 ± 5^bc^	−3.1 ± 0.6^d^	19 ± 2^a^
E2	CM:PPI (85:15)-M15	0.13 ± 0.01^c^	11.5 + 1.2^a^	8.9 ± 1.8^abc^	31 ± 2^a^	47 ± 5^c^	−0.9 ± 0.3^c^	14 ± 2^bc^
E3	CM:PPI (70:30)-M15	0.28 ± 0.01^bc^	7.6 + 1.2^c^	9.4 ± 2.2^a^	31 ± 2^ab^	48 ± 4^c^	1.0 ± 0.4^b^	14 ± 3^bc^
E4	CM:PPI (50:50)-M17	0.37 ± 0.02^bc^	5.5 + 0.7^d^	9.0 ± 1.3^ab^	26 ± 2^cd^	48 ± 3^c^	2.3 ± 0.4^a^	14 ± 2^bc^
E5	CM:PPI (50:50)-M16	0.35 ± 0.02^bc^	5.7 + 0.6^d^	7.1 ± 0.8^bc^	26 ± 3^cd^	51 ± 2^bc^	2.7 ± 0.6^a^	15 ± 2^b^
E6	CM:PPI (50:50)-M15	0.44 ± 0.02^b^	5.2 + 0.6^d^	9.0 ± 1.2^ab^	27 ± 2^cd^	54 ± 3^ab^	3.1 ± 1.0^a^	14 ± 3^bc^
E7	CM:PPI (30:70)-M17	1.28 ± 0.12^a^	3.5 + 0.1^e^	2.8 ± 0.6^d^	24 ± 2^d^	59 ± 3^a^	3.0 ± 0.9^a^	13 ± 1^bc^
E8	CM:PPI (30:70)-M16	1.45 ± 0.12^a^	3.6 + 0.2^e^	2.9 ± 0.4^d^	24 ± 2^cd^	59 ± 4^a^	2.6 ± 1.1^a^	12 ± 2^c^
E9	CM:PPI (30:70)-M15	1.32 ± 0.26^a^	3.6 + 0.4^e^	2.7 ± 0.8^d^	24 ± 2^cd^	58 ± 4^a^	3.2 ± 1.5^a^	13 ± 2^bc^

⁎ The values within the same column followed by the same letters are not significantly different (*p =* 0.05) according to Tukey's test.

# E: extrudate, CM: cornmeal, PPI: pea protein isolate, M: moisture content.

Lightness (L*) remained unchanged with up to 50% pea protein isolate in the feed, after which it increased significantly (*p <* 0.05). Redness (a*) showed a consistent and significant increase with rising pea protein isolate levels in the feed. In contrast, yellowness (b*) decreased significantly with the addition of 15% pea protein isolate and exhibited no further change at higher levels. Chroma (C*) values were higher in the corn-only extruded sample, indicating a more vivid colour, while samples containing pea protein isolate showed duller tones, although differences among these samples were not significant (*p >* 0.05). At low substitution levels (0–15% PPI), hue angle (*h*°) values remained high, indicating a stable yellow coloration characteristic of corn-rich formulations. A pronounced decrease in hue angle was observed at 30% PPI, indicating a major transition in colour characteristics and the onset of browning reactions. Further increases in PPI content of the feed (50–70%) did not change hue angle significantly (*p >* 0.05), suggesting a plateau in colour development. Moisture content, on the other hand, had no effect on the colour parameters of the extrudates. It was reported that increasing quinoa incorporation in corn extrudates decreased L* values due to the Maillard reaction and quinoa's inherently darker pigments. Increasing quinoa levels also led to reduced b* values, attributed to carotenoid degradation and pigment instability under thermomechanical stress, while a* values shifted toward negative because of quinoa colour and phenolic compounds (Filipović et al., 2026). It is difficult to distinguish the individual effects of pea protein isolate addition and extrusion cooking on colour, because pea protein itself has a pale-yellow colour, and multiple chemical reactions, such as the Maillard reaction, caramelisation, and the degradation of colour pigments, occur simultaneously during extrusion (Yi et al., 2022). However, colour is not only the result of chemical reactions. It was also reported previously that higher L* values of the puffed products might be related to expansion in the die exit which contributes to increased surface area of the product and the higher dispersion of light (Paes & Joseph, 2004). Therefore, colour values of extruded products can be explained by both physical structure of the product and chemical reactions.

Processing parameters measured under different screw speeds (400, 450, and 500 rpm) and barrel temperature profiles (T1: up to 145 °C, T2: up to 150 °C, T3: up to 155 °C, and T4: up to 160 °C) for the extrusion of cornmeal-pea protein isolate feeds with compositions of 50:50 and 30:70 are presented in Table 3. A decrease in screw speed resulted in higher torque and lower specific mechanical energy (SME), while the feed rate remained constant. This indicates that reduction in screw speed outweighs the increase in torque, resulting in a decrease in SME. Because SME is directly proportional to both screw speed and torque and inversely proportional to the feed rate (Lyu et al., 2022; Ribeiro et al., 2024). Lower SME values were noted also by Attenborough et al. (2023) by reducing screw speed of extruder during processing of corn grit. Similarly, the effect of screw speed on shear and mechanical energy has been reported for texturized vegetable proteins composed of soy protein isolate, wheat gluten and corn starch, where lowering the screw speed resulted in a reduction in specific mechanical energy (Lyu et al., 2022). Increasing the barrel temperature profile (T1 to T4) elevated the melt temperature, lower torque value and SME. In parallel to findings here, Kesre and Masatcioglu (2022) reported that increasing die-temperature causes a decrease in viscosity resulting in decreases in the torque and SME values during extrusion of corn flour- red lentil bran.

**Table 3:** Process and material characteristics during extrusion of cornmeal-pea protein isolate feed compositions (50:50 and 30:70) at varying barrel temperature profiles and screw speeds⁎#.

	E10	E11	E6	E12	E13	E14	E15	E16	E9	E17	E18	E19
	CM:PPI (50:50)-SS500	CM:PPI (50:50)-SS450	CM:PPI (50:50)-SS400	CM:PPI (50:50)- T2	CM:PPI (50:50)- T3	CM:PPI (50:50)- T4	CM:PPI (30:70)-SS500	CM:PPI (30:70)-SS450	CM:PPI (30:70)-SS400	CM:PPI (30:70)- T2	CM:PPI (30:70)- T3	CM:PPI (30:70)- T4
Bulk Density of Dry Feed (g/mL)	0.49± 0.003	0.49± 0.003	0.49± 0.003	0.49± 0.003	0.49± 0.003	0.49± 0.003	0.49± 0.005	0.49± 0.005	0.49± 0.005	0.49± 0.005	0.49± 0.005	0.49± 0.005
Moisture Content of Dry Feed (%)	3.3	3.3	3.3	3.3	3.3	3.3	3.45	3.45	3.45	3.45	3.45	3.45
Moisture Content of Extrudate (%)	9.1 ± 0.4	8.7 ± 0.5	9.9 ± 0.8	9.4 ± 0.5	9.4 ± 0.4	9.3 ± 0.5	9.5 ± 0.1	9.9 ± 0.5	9.7 ± 0.4	8.9 ± 0.7	8.7 ± 0.6	9.0 ± 0.5
Pressure (kPa)	4800	4800	4800	5000	4500	4300	4500	5000	4200	4700	3500	3200
Torque (Nm)	12.2	13.3	14.4	14.0	12.9	12.2	11.5	12.6	13.3	12.2	11.0	10.0
Melt Temperature (°C)	153	152	152	152	157	161	147	147	147	152	156	161
Screw Speed (rpm)	500	450	400	400	400	400	500	450	400	400	400	400
Specific Mechanical Energy (kJ/kg)	648	648	540	540	540	540	612	576	540	504	468	432
Barrel Temperature Profile (°C)												
Zone 1	40	40	40	40	40	40	40	40	40	40	40	40
Zone 2	60	60	60	60	60	60	60	60	60	60	60	60
Zone 3	80	80	80	80	80	80	80	80	80	80	80	80
Zone 4	100	100	100	110	110	110	100	100	100	110	110	110
Zone 5	120	120	120	130	135	140	120	120	120	130	135	140
Zone 6	140	140	140	145	150	155	140	140	140	145	150	155
Zone 7	145	145	145	150	155	160	145	145	145	150	155	160
Zone 8	145	145	145	150	155	160	145	145	145	150	155	160

⁎ Moisture content of feed (%): 15, flow rate of liquid feed (mL/min): 8, and feed rate of dry feed (kg/h): 3.4 were same for each extrusion.

# E: extrudate, CM: cornmeal, PPI: pea protein isolate, SS: screw speed, T: temperature profile.

Bulk density of the extrudates was mostly not affected by screw speed (except for 30:70 CM:PPI at 500 rpm in comparison to 450 rpm) or barrel temperature profile under the conditions applied here (Table 4). Diameter also remained unchanged under all tested extrusion conditions. An increase in the bulk density of lentil protein isolate at constant moisture content was reported with either increasing screw speed from 300 to 450 rpm or increasing extrusion temperature from 120 to 140 °C (Singh et al., 2024). Screw speed was also reported to have a significant effect (*p* < 0.05) on bulk density although no significant effects were noted with the change in the die temperature during extrusion of low-starch extruded animal feed containing *Clostridium autoethanogenum* protein (Ma et al., 2023).

**Table 4:** Physical properties of cornmeal-pea protein isolate extrudates (50:50 and 30:70 blends) at varying screw speeds and barrel temperature profiles⁎, #.

		Bulk Density (g/mL)	Diameter (mm)	Hardness (N)	Fracturability (mm)	*L*⁎	*a*⁎	*b*⁎
E10	CM:PPI (50:50)-SS500	0.42 ± 0.02^c^	5.0 + 0.6^a^	7.7 ± 1.0^ab^	25 ± 3^ab^	51 ± 1^d^	3.0 ± 0.5^ab^	15 ± 1^abc^
E11	CM:PPI (50:50)-SS450	0.39 ± 0.02^c^	5.1 + 0.4^a^	8.0 ± 1.3^ab^	26 ± 3^ab^	52 ± 2^cd^	3.1 ± 1.2^ab^	14 ± 3^abcd^
E6	CM:PPI (50:50)-SS400	0.44 ± 0.02^c^	5.2 + 0.6^a^	9.0 ± 1.2^a^	27 ± 2^ab^	54 ± 3^bcd^	3.1 ± 1.0^ab^	14 ± 3^abcd^
E12	CM:PPI (50:50)-T2	0.43 ± 0.02^c^	5.1 + 0.6^a^	7.5 ± 0.8^b^	27 ± 1^a^	53 ± 2^bcd^	3.5 ± 1.2^a^	15 ± 2^a^
E13	CM:PPI (50:50)-T3	0.43 ± 0.02^c^	5.3 + 0.7^a^	7.8 ± 1.4^ab^	26 ± 2^ab^	53 ± 1^bcd^	2.9 ± 1.0^ab^	14 ± 3^abcde^
E14	CM:PPI (50:50)-T4	0.44 ± 0.03^c^	5.1 + 0.6^a^	7.0 ± 1.0^b^	26 ± 3^ab^	52 ± 3^bcd^	3.3 ± 1.0^ab^	15 ± 2^ab^
E15	CM:PPI (30:70)-SS500	1.53 ± 0.16^a^	3.6 + 0.2^b^	2.5 ± 0.4^c^	24 ± 2^b^	56 ± 1^abc^	3.7 ± 0.4^a^	13 ± 1^abcde^
E16	CM:PPI (30:70)-SS450	1.10 ± 0.06^b^	3.8 + 0.3^b^	2.7 ± 0.6^c^	24 ± 2^ab^	56 ± 4^ab^	3.0 ± 0.7^ab^	12 ± 1^bcde^
E9	CM:PPI (30:70)-SS400	1.32 ± 0.26^ab^	3.6 + 0.4^b^	2.7 ± 0.8^c^	24 ± 2^ab^	58 ± 4^a^	3.2 ± 0.5^ab^	13 ± 2^abcde^
E17	CM:PPI (30:70)-T2	1.48 ± 0.10^a^	3.4 + 0.3^b^	2.2 ± 0.5^c^	24 ± 2^ab^	59 ± 3^a^	2.5 ± 0.9^ab^	12 ± 1^cde^
E18	CM:PPI (30:70)-T3	1.40 ± 0.11^a^	3.7 + 0.5^b^	2.2 ± 0.7^c^	25 ± 1^ab^	60 ± 2^a^	2.4 ± 0.6^ab^	12 ± 1^de^
E19	CM:PPI (30:70)-T4	1.27 ± 0.10^ab^	3.6 + 0.4^b^	2.5 ± 0.7^c^	24 ± 2^ab^	60 ± 4^a^	2.0 ± 0.8^b^	11 ± 2^e^

⁎ The values within the same column followed by the same letters are not significantly different (*p =* 0.05) according to Tukey's test.

# E: extrudate, CM: cornmeal, PPI: pea protein isolate, SS: screw speed, T: temperature profile.

Screw speed did not affect hardness significantly (*p* > 0.05). Hardness of extrudates decreased significantly (*p <* 0.05) with increasing barrel temperature from the initial settings to T1 and no further decrease was noted with further increase in temperature (T2, T3, T4) when the 50:50 cornmeal-pea protein isolate feed was used. However, no significant difference (*p* > 0.05) in hardness was observed when the pea protein content in the feed was increased to 70% with changing temperature. A negative correlation was reported between hardness and temperature and between hardness and screw speed by Jacquet et al. (2025) due to decrease in viscosity. The reduction in hardness with increasing barrel temperature may be associated with a more porous extrudate structure (50,50 cornmeal,pea protein isolate), likely linked to starch content, since increasing protein isolate levels (30,70 cornmeal,pea protein isolate) did not result in significant changes (*p >* 0.05). Guo et al. (2025) also reported that starch promotes the expansion of peanut protein extrudates and increases pore size and porosity in the structure, hence reduces hardness. Fracturability was not influenced by any of the processing parameters. Similarly, colour values were unaffected by screw speed or temperature profile under the tested conditions. Previous studies have reported that increasing temperature decreases L* values of extrudates due to the Maillard reaction, whereas higher screw speeds limit browning because of reduced residence time (Pismag et al., 2024; Usman et al., 2026). Overall, neither screw speed nor barrel temperature profile has any effect on physical quality characteristics of extrudates, except for hardness, under the tested conditions. However, more prominent changes in the screw speed and/or temperature may reveal more pronounced effects on the physical properties of protein-enriched extrudates.

Scale-up of twin-screw extrusion cannot be achieved by directly transferring operating conditions such as screw speed or feed rate. However, these parameters can help explain the fundamental aspects of product quality. Instead, scale-up requires reproducing scale-independent parameters. In this context, specific mechanical energy, temperature, residence time distribution, and the overall thermal history experienced by the material are of importance. An efficient twin-screw extrusion model based on a continuum-mechanics approach, capable of predicting fundamental process parameters and their response to changes in control variables, can therefore be a valuable tool for industrial design. Nevertheless, defining product quality remains challenging, as quality attributes are not always easily expressed in terms of measurable structural features (Della Valle et al., 2012).

### 3.2 Acrylamide content of cornmeal-pea protein isolate extrudates

**Fig. 1:**
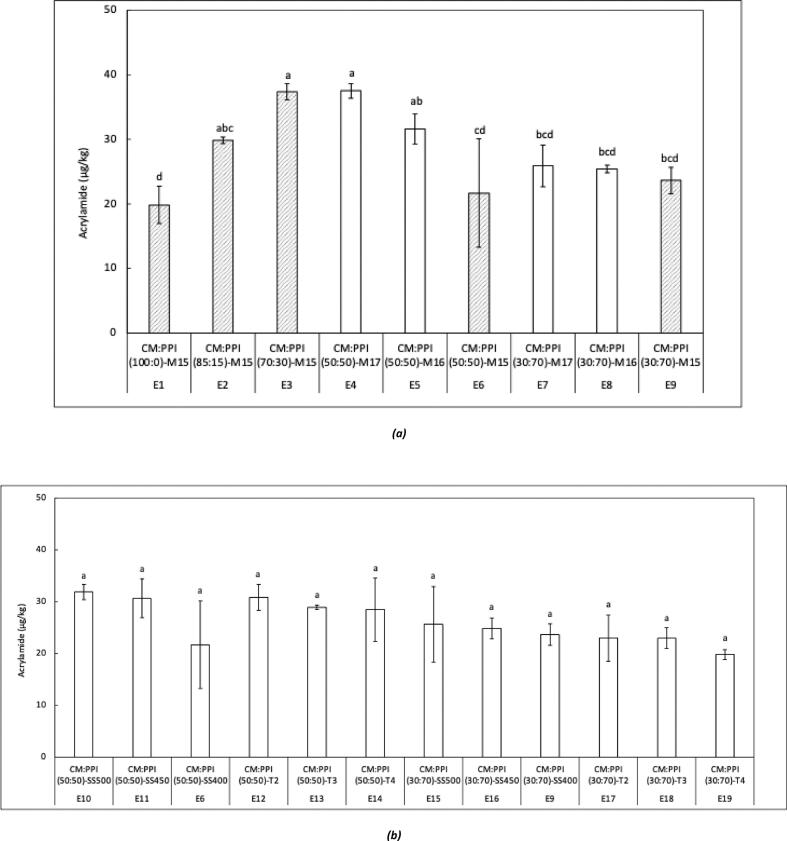
Acrylamide content of cornmeal-pea protein isolate extrudates **(a)** with different compositions at varying moisture contents **(b)** (50,50 and 30,70 blends) at varying screw speeds and barrel temperature profiles. E1: CM:PPI(100:0)-M15; E2: CM:PPI(85:15)-M15; E3: CM:PPI(70:30)-M15; E4: CM:PPI(50:50)-M17; E5: CM:PPI(50:50)-M16; E6: CM:PPI(50:50)-M15-SS400; E7: CM:PPI(30:70)-M17; E8: CM:PPI(30:70)-M16; E9:CM:PPI(30:70)-M15-SS400; E10: CM:PPI(50:50)-SS500; E11: CM:PPI(50:50)-SS450; E12: CM:PPI(50:50)-T2; E13: CM:PPI(50,50)-T3; E14: CM:PPI(50,50)-T4; E15: CM:PPI(30:70)-SS500; E16: CM:PPI(30:70)-SS450; E17: CM:PPI(30:70)-T2; E18: CM:PPI(30,70)-T3; E19: CM:PPI(30,70)-T4; E: extrudate; CM: cornmeal; PPI: pea protein isolate; M: moisture content; SS: screw speed; T: temperature profile.

Acrylamide contents of the cornmeal and pea protein isolate were found to be 2.1 ± 0.2 and 12.2 ± 1.5 μg/kg, respectively. The presence of acrylamide in these raw materials can be attributed to conditions to which they were exposed during drying process. Acrylamide contents of cornmeal-pea protein isolate extrudates produced from aforementioned raw materials under varying feed compositions and moisture levels are presented in Fig. 1a. The addition of 15% pea protein isolate significantly (*p <* 0.05) increased the acrylamide content from 19.8 ± 2.9 to 37.4 ± 1.3 μg/kg. However, further increases in pea protein content to 30%, 50%, and 70% did not cause additional increases. In fact, the acrylamide level decreased when 50% and 70% pea protein isolate was mixed with cornmeal compared with the 15% pea protein isolate containing formulation. Incorporation of pea protein isolate did not elevate the asparagine level of the feed, as its asparagine content (15 mg/kg) was much lower than that of cornmeal (128 mg/kg) (**Supplementary Material, Table S2**). Similarly, the reducing sugar (0.011%) and total sugar (0.031%) contents of pea protein isolate were lower than those of cornmeal (0.4% and 1.0%, respectively). Additionally, the total free amino acid content of the cornmeal and pea protein isolate were not significantly different, hence, the competition effect of other free amino acids over asparagine for sugar is also negligible. Therefore, the initial increase in acrylamide formation cannot be attributed to asparagine or sugar content of pea protein isolate. Instead, it may be related to lipid oxidation products with reactive carbonyl groups from pea protein isolate, which can promote Maillard reaction in the presence of asparagine from cornmeal. At higher pea protein isolate levels (50–70%), the dilution of asparagine likely limited acrylamide formation despite the potential presence of such reactive compounds. Explaining acrylamide formation in the context of changing feed composition, including variations in asparagine, reducing sugars, and lipid oxidation products, is challenging. Therefore, model system studies may be more appropriate for elucidating the underlying mechanism. Notably, the acrylamide levels in all samples were well below the benchmark value of European Commission for non-whole grain or non-bran-based cereals (150 μg/kg) (EC European Commission, 2017). Although lower moisture generally favours the Maillard reaction, no significant differences (*p >* 0.05) in acrylamide content were observed within the tested moisture range (15–17%). Similarly, increasing screw speed or barrel temperature (Fig. 1b) did not significantly (*p >* 0.05) affect acrylamide formation, likely due to the limited asparagine content in the 50:50 and 30:70 cornmeal-pea protein isolate formulations.

Acrylamide was reported to change from <LOQ to 210 μg/kg under low SME conditions set-up (96–243 Wh/kg) during extrusion cooking of breakfast cereals with corn semolina with an initial moisture content of 11.7 to 20% and barrel temperature of 180 °C (Lipinski et al., 2025). The acrylamide values were higher than reported than in this study. Jozinović et al. (2024) incorporated a variety of defatted press cakes into corn grits to increase the protein content of extruded products, processed either directly or with the injection of supercritical CO₂, and reported that acrylamide levels in the extrudates were below the limit of detection (< 3.79 μg/kg), suggesting that extrusion is a safe processing method.

### 3.3 Aroma profile of cornmeal, pea protein isolate and cornmeal-pea protein extrudates

#### 3.3.1 Effect of feed composition and moisture content

Aroma compounds in pea protein isolate and cornmeal before extrusion were mainly derived from Maillard reactions, amino acid degradation, and lipid oxidation, with no pyrazines detected (**Supplementary Material, Table S3**). In total, 42 selected volatile compounds were identified and semi-quantified in pea protein isolate. Among them, the content of lipid-derived aldehydes, ketones, alcohols, and acids was particularly high in pea protein isolate. The presence of hexanal, 2-pentylfuran, hexanoic acid, benzaldehyde, (*E,Z*)-3,5-octadien-2-one, 1-octen-3-ol, and nonanal were remarkable because they were relatively high compared to others. The presence and abundance of these volatiles can be attributed to the pre-extrusion processing. Pea protein isolate is typically obtained through wet milling, during which lipoxygenase enzymes become activated, followed by a drying step that further promotes lipid oxidation (Tuncel et al., 2024). Ebert et al. (2022) identified hexanal, nonanal, 2-undecanone, (*E*)-2-octenal, (*E,Z*)-3,5-octadien-2-one, (*E,E*)-2,4-decadienal, 2-pentylfuran, 2-pentylpyridine, and γ-nonalactone as major off-flavour contributors in pea protein isolates. Similarly, hexanal, (*E*)-2-octenal, nonanal, 1-octen-3-ol, and 2-pentylfuran have been consistently reported as common lipid oxidation products in pea flour, pea protein solutions, pea protein beverages, and pea milk (Bi et al., 2020; Leonard et al., 2023; Trindler et al., 2022; el Youssef et al., 2020). Li et al. (2024b) determined the odour activity values of aroma compounds in pea protein isolate and reported that hexanal, nonanal, benzaldehyde, (*E,E*)-2,4-decadienal, 1-octen-3-ol, and 2-pentylfuran exhibited the highest flavour dilution (FD) factors (FD = 243). Among them, (*E,E*)-2,4-decadienal was reported with the highest odour activity value (26431).

**Fig. 2:**
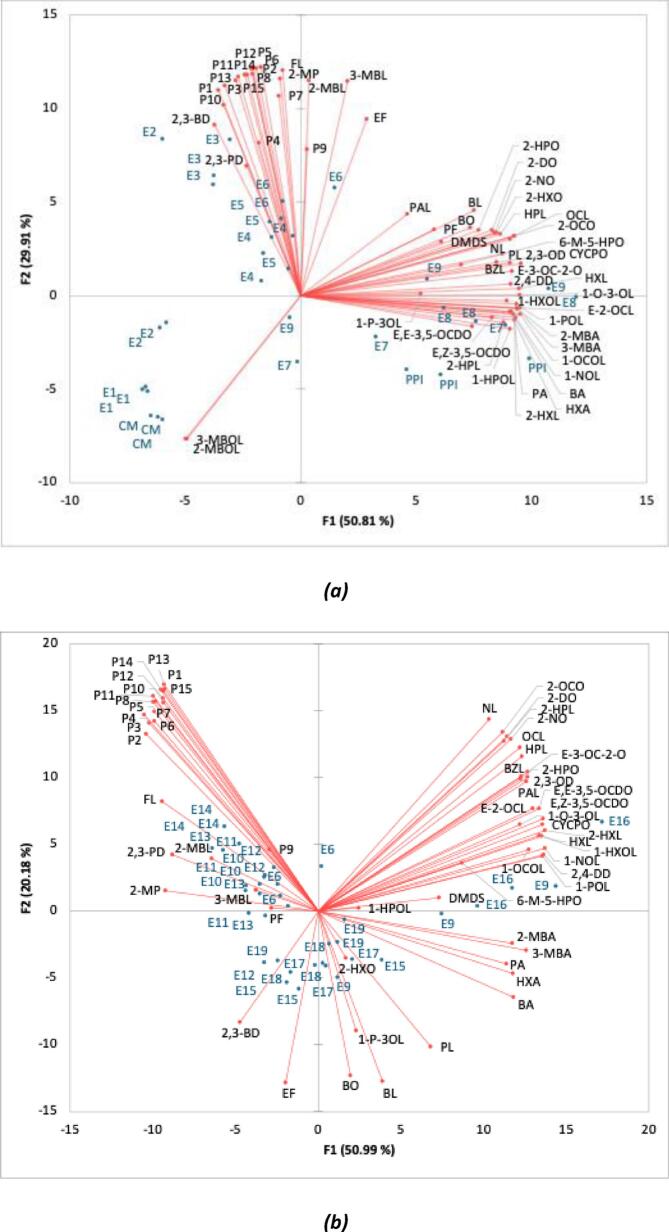
PCA plot of aroma compounds of cornmeal-pea protein isolate extrudates **(a)** with different compositions at varying moisture contents and their raw materials **(b)** (50:50 and 30:70 blends) at varying screw speeds and barrel temperature profiles. Strecker aldehydes (2-MP: 2-methylpropanal, 3-MBL: 3-methylbutanal, 2-MBL: 2-methylbutanal, PAL: phenylacetaldehyde), lipid-derived aldehydes (BL: butanal, PL: pentanal, HXL: hexanal, 2-HXL: 2-hexenal, HPL: heptanal, 2-HPL: (E)-2-heptenal, OCL: octanal, E-2-OCL: (E)-2-octenal, NL: nonanal, (E,E)-2,4-decadienal: 2,4-DD), other aldehydes (BZL: benzaldehyde, FL: 2-furfural), diketones (2,3-butanedione, 2,3-PD: 2,3-pentanedione, 2,3-OD: 2,3-octanedione), ketones (BO: 2-butanone, 2-HXO: 2-hexanone, 2-HPO: 2-heptanone, 6-M-5-HPO: 6-methyl-5-hepten-2-one, 2-OCO: 2-octanone, CYCPO: 2,3-dimethyl-2-cyclopenten-1-one, E-3-OC-2-O: (E)-3-octen-2-one, E,E-3,5-OCDO: (E,E)-3,5-octadien-2-one, 2-NO: 2-nonanone, E,Z-3,5-OCDO: (E,Z)-3,5-octadien-2-one, 2-DO: 2-decanone), alcohols (1-P-3OL: 1-penten-3-ol, 3-MBOL: 3-methylbutanol, 2-MBOL: 2-methylbutanol, 1-POL: 1-pentanol, 1-HXOL: 1-hexanol, 1-HPOL: 1-heptanol, 1-O-3-OL: 1-octen-3-ol, 1-OCOL: 1-octanol, 1-NOL: 1-nonanol), furans (EF: 2-ethylfuran, PF: 2-pentylfuran), sulfur compounds (DMDS: dimethyl disulfide), acids (BA: butanoic acid, 3-MBA: 3-methylbutanoic acid, 2-MBA: 2-methylbutanoic acid, PA: pentanoic acid, HXA: hexanoic acid), pyrazines (P1: pyrazine, P2: 2-methylpyrazine, P3: 2,5(6)-dimethylpyrazine, P4: 2-ethylpyrazine, P5: 2,3-dimethylpyrazine, P6: 2-ethyl-5-methylpyrazine, P7: 2-ethyl-6-methylpyrazine, P8: trimethylpyrazine, P9: 2-ethyl-3-methylpyrazine, P10: 2-ethenyl-6-methylpyrazine, P11: 3-ethyl-2,5-dimethylpyrazine, P12: 2-methyl-6-1-propenylpyrazine, P13: 2,3-diethyl-5(or 6)-methylpyrazine, P14: 2-methyl-3,5-diethylpyrazine, P15: 3-propyl-2,5-dimethylpyrazine).

A total of 26 volatile compounds were identified and semi-quantified in cornmeal (**Supplementary Material, Table S3**). Although the number of volatile compounds was lower than in pea protein isolate, cornmeal processing similarly involves water immersion and drying steps during separation or milling, which can promote lipid oxidation and the formation of Maillard-derived volatile compounds (Gwirtz & Garcia-Casal, 2014). Unlike pea protein isolate, cornmeal contained 2-methylbutanal, 2-methylbutanol, and 3-methylbutanol. 2-Methylbutanal can be formed via Strecker degradation of isoleucine, whereas 2-methylbutanol and 3-methylbutanol likely originated from the reduction of the corresponding Strecker aldehydes (2- and 3-methylbutanal) or from enzymatic activity occurring during kernel drying (Smit et al., 2009). After extrusion, the cornmeal extrudate contained 2-methylbutanal and 2- and 3-methylbutanol at levels not significantly different (*p >* 0.05) than from those in the raw cornmeal, indicating that under the applied extrusion conditions, their formation was not promoted, or they may have evaporated during sudden expansion. In the PCA plot (Fig. 2a), 2-methylbutanol and 3-methylbutanol were clearly separated among the samples, distinguishing cornmeal (CM), the cornmeal extrudate (E1), and the extrudate containing 15% pea protein isolate (E2) from the other samples. Extrudates containing 30 and 50% of pea protein isolate in their feed were clustered together (E3 - E6) and were mainly associated with Maillard reaction products, whereas extrudates with 70% pea protein isolate in its feed (E7 - E9) clustered with pea protein isolate (PPI), and were associated with lipid oxidation products. Changes in moisture content did not noticeably affect the distribution of the compounds or sample groupings.

2-Methylpropanal, the Strecker aldehyde of valine, was not detected in the raw materials but was formed during extrusion of cornmeal. Its concentration increased when 30% of pea protein isolate was incorporated into feed but did not change further with higher levels of incorporation. When the pea protein isolate concentration in the feed reached 70%, a decrease in 2-methylpropanal was found. Similarly, 3-methylbutanal and 2-methylbutanal, Strecker aldehydes of leucine and isoleucine, respectively, followed the same trend as 2-methylpropanal. At moderate pea protein isolate addition level (30%), the increased amino acid content in the feed, together with reducing sugars from cornmeal (present at higher levels than in pea protein isolate), promoted the formation of Strecker aldehydes. However, when pea protein isolate levels in the feed exceeded 50%, further increases in protein content did not lead to higher Strecker aldehyde formation. This may be attributed to changes in the balance of precursors involved in Strecker degradation. While concentrations of amino acids increased with pea protein incorporation, the proportion of cornmeal, the main source of reducing sugars, decreased. Therefore, the availability of sugar-derived intermediates potentially involved in Strecker degradation may have been limited at higher protein substitution levels. In contrast to those Strecker aldehydes, phenylacetaldehyde increased upon extrusion but did not vary significantly (*p* > 0.05) with changes in feed composition.

Pentanal, hexanal, heptanal, (*E*)-2-heptenal, octanal, (*E*)-2-octenal, nonanal, (*E,E*)-2,4-decadienal were found as common lipid-derived aldehydes in both cornmeal and pea protein isolate. In addition, pea protein isolate was found to contain butanal and (*E*)-2-hexenal, and the concentrations of these lipid-derived aldehydes were significantly higher than those in cornmeal. Consequently, increasing pea protein isolate in the feed led to higher levels of lipid oxidation-derived aldehydes, which originated primarily from the raw material itself rather than being formed during extrusion. Lipid oxidation-derived aldehydes in pea protein concentrates and isolates were reported previously (Liu et al., 2023). The presence of these compounds in the raw material might be related to the any stage of the plant maturation, harvesting, processing or storage (Esmi et al., 2025).

2-Furfural, a common product of sugar degradation and the Maillard reaction, reached its maximum concentration when 50% pea protein isolate was incorporated into the feed, with levels approximately five times higher than those in the cornmeal-only extrudate. Benzaldehyde, which imparts a nutty, almond-like aroma, originated from the pea protein isolate, and its concentration increased proportionally with pea protein addition to the feed. Presence of benzaldehyde in pea protein isolate can be attributed to free radical initiated oxidative cleavage of the carbon‑carbon double bond of the enolized phenylacetaldehyde (Fong & Yaylayan, 2008).

2,3-Butanedione was primarily formed through Maillard reaction during extrusion, and its concentration was not affected by increasing pea protein isolate levels in the feed. In contrast, 2,3-pentanedione, which originated mainly from pea protein isolate, showed a decrease in concentration when the pea protein isolate content in the feed reached 70%. Hence, both 2,3-butanedione and 2,3-pentanedione were found to be correlated with the lower pea protein isolate content extrudates (E3 - E6) in the PCA plot (Fig. 2a). 2,3-Octanedione was derived from both cornmeal and pea protein isolate, but its concentration was not significantly (*p >* 0.05) affected by changes in feed composition and found to be related to higher pea protein isolate containing extrudates (E7 - E9) (**Supplementary Material, Table S3**).

A wide range of ketones were detected in pea protein isolate, predominantly originating from lipid oxidation, including 2-butanone, 2-hexanone, 2-heptanone, 2-octanone, (*E*)-3-octen-2-one, (*E,E*)-3,5-octadien-2-one, 2-nonanone, (*E,Z*)-3,5-octadien-2-one, and 2-decanone. In contrast, 6-methyl-5-hepten-2-one has been linked to carotenoid degradation, while the formation pathway of 2,3-dimethyl-2-cyclopenten-1-one is not well established. Cornmeal contained only 2-heptanone and 6-methyl-5-hepten-2-one. During extrusion, the concentrations of 2-hexanone and 2-heptanone increased significantly (*p* < 0.05) when the pea protein isolate content in the feed was raised to 50%. Similarly, 2,3-dimethyl-2-cyclopenten-1-one increased markedly when the pea protein isolate level in the feed reached 70%. All these ketones were clustered together with high pea protein isolate containing extrudates.

Seven alcohols, 1-penten-3-ol, 1-pentanol, 1-hexanol, 1-heptanol, 1-octen-3-ol, 1-octanol, and 1-nonanol, were identified in pea protein-enriched extrudates. Their concentrations increased with higher pea protein isolate content in the feed, despite the expectation that volatile alcohols might evaporate during the extrusion and expansion processes. The presence of these alcohols was strongly associated with formulations containing high levels of pea protein (samples E6 - E9). This might be attributed to the lipid oxidation in the pea protein during the isolation process.

In terms of furans, 2**-**ethylfuran was detected exclusively in pea protein isolate, while 2-pentylfuran originated from both cornmeal and pea protein isolate. Both compounds showed increasing concentrations with higher pea protein isolate levels in the feed. However, the 2-ethylfuran content in the extruded extrudates did not exceed that of the raw pea protein isolate, suggesting that the extrusion process did not promote additional lipid oxidation or it evaporated from the die exit. On contrary, 2-pentylfuran was reported to increase during low-moisture extrusion of oat flour (Zhang et al., 2023).

Dimethyl disulfide was detected in both cornmeal and pea protein isolate. Although its concentration approximately doubled in extrudates with high pea protein content (70% in the feed), this increase was not statistically significant (*p* > 0.05).

Short-chain fatty acids (≤ C6) were present in pea protein isolate, whereas cornmeal had no detectable acids except for hexanoic acid**.** Among the identified acids, hexanoic acid was the most abundant, followed by pentanoic acid**.** The extrusion process did not promote the formation of additional acids, as their concentrations in the final extrudates did not exceed those measured in the raw pea protein isolate.

A total of fifteen pyrazines were identified in pea protein-enriched extrudates, whereas none were detected in the raw materials, indicating that these compounds were formed during the extrusion process. The cornmeal-only extrudate contained nine pyrazines, and their concentrations were the lowest among all formulations. Incorporation of 15% pea protein isolate into the feed promoted the formation of pyrazines such as 2-ethyl-3-methylpyrazine, 2-methyl-6-(1-propenyl)pyrazine, 2-methyl-3,5-diethylpyrazine, and 3-propyl-2,5-dimethylpyrazine. The highest total pyrazine was achieved when 30% pea protein isolate was added to the feed. Among the identified compounds, 3-ethyl-2,5-dimethylpyrazine was notable in the extrudates. This compound contributes a characteristic nutty aroma and has an exceptionally low odour threshold (0.4 μg/L; (van Gemert, 2011)). Another abundant pyrazine was 2,5(6)-dimethylpyrazine, which is not that odour active compared to 3-ethyl-2,5-dimethylpyrazine but imparts roasted, nutty, and chocolate-like notes, with reported odour thresholds of 800 μg/L and 200 μg/L in water for 2,5-dimethylpyrazine and 2,6-dimethylpyrazine, respectively (van Gemert, 2011). Trimethylpyrazine is associated with toasted, nutty, chocolatey, and coffee-like aroma notes and has an odour threshold of 400 μg/L in water (van Gemert, 2011). 2-Ethyl-3-methylpyrazine appeared after the addition of 15% pea protein isolate and remained relatively constant with further increases in pea protein isolate concentration. The presence of this compound is noteworthy for its low odour threshold (0.4 μg/L; (van Gemert, 2011)) and its contribution to burnt, roasted, nutty, and cereal aroma characteristics. Overall, increasing the pea protein isolate concentration in the feed up to 30% enhanced pyrazine formation; however, no further increase was observed beyond this level, and a decrease occurred when the PPI content reached 70%. *At intermediate protein inclusion (30%), reducing sugars from cornmeal combined with increasing amino acid from pea protein isolate may have favoured pyrazine formation. In contrast, at higher protein substitution (70%), the substantial reduction in cornmeal content may have limited the availability of sugar-derived intermediates required for Maillard reactions, despite increased amino acid levels. As a result, pyrazine formation may have become sugar-derived carbonyl-limited at high protein levels.* Pyrazines in PCA plot were clustered with 30 and 50% pea protein isolate containing samples (E3 - E6) (Fig. 2a).

#### 3.3.2 Effect of screw speed and temperature profile

The volatile compounds separated well in terms of Maillard reaction products (left-upper side) and lipid oxidation products (right and left-down side) in the PCA plot (Fig. 2b), which were found to be more related to feed composition, (E10, E11, E6, E12, E14 having 50%; E15, E16, E9, E17, E18, E19 having 70% pea protein isolate in the feed) rather than screw speed or temperature profile. An increase in screw speed is associated with lower mechanical shear and shorter residence time (Pismag et al., 2024), which in turn reduces the thermal load on the material. Consequently, from the perspective of screw speed and chemical reactions, slower screw speeds would be expected to promote a greater extent of Maillard reactions, sugar degradation, and/or lipid oxidation. In line with this expectation, a significant (*p* < 0.05) increase in ketones and aldehydes was observed with decreasing screw speed (E15, E16, and E9, respectively) in extrudates formulated with 50% pea protein isolate. This effect may be attributed to enhanced lipid oxidation resulting from the longer residence time at lower screw speeds. In contrast, there was no change in pyrazine levels depending on the screw speed in the extrudates having 70% pea protein isolate in their feed (**Supplementary Material, Table S4**). Moreover, no significant (*p* > 0.05) change was found in any aroma compounds of extrudates when the feed had 50% pea protein isolate (E10, E11, E6). The increase in lipid oxidation products in the extrudates may be attributed to and the degradation of pre-existing lipid hydroperoxides during extrusion. This phenomenon became pronounced when the pea protein isolate content in the formulation was increased to 70%.

The increases in the barrel temperature zones affected only pyrazines, not the other volatile compounds (**Supplementary Material, Table S4**) hence there was no obvious separation among the samples depending on the temperature (Fig. 2b). Increase in barrel temperature profile increased pyrazines independent of the composition. However, the level of pyrazines was also dependent on the composition of the feed as they form through Maillard reaction by the reaction of sugars and free amino acids. Extrudates produced by incorporating 70% pea protein isolate into the feed in place of cornmeal exhibited lower pyrazine levels than those produced with 50% pea protein isolate under all temperature conditions evaluated. The reason for that could be the dilution of feed in terms of sugars when pea protein isolate incorporated more. The most pyrazine rich extrudate was with cornmeal-pea protein isolate (50,50, *w/w*) feed composition, screw speed of 400 rpm, and produced with the temperature profile of the eight zones of 40–60–80–110–140–155–160–160 °C. By increasing temperature of the zones of the barrel, from 40 to 60–80–100–120–140–145–145 °C (T1) to 40–60–80–110–140–155–160–160 °C (T4), the level of pyrazines (2-methylpyrazine, 2,5(6)-dimethylpyrazine, 2-ethylpyrazine, 2,3-dimethylpyrazine, 2-ethyl-5-methylpyrazine, trimethylpyrazine, 3-ethyl-2,5-dimethylpyrazine, 2-methyl-3,5-dimethylpyrazine) nearly doubled. However, no significant increases in pyrazines were found when the intermediate temperature profiles were applied (T2 and T3). It was reported that the reason for the perceived differences in extruded whole grain maize was associated with increased levels of Maillard reaction products, such as 2-ethyl-3,5-dimethylpyrazine and 2-acetyl-2-thiazoline (Smith & Peterson, 2020).

## 4 Conclusion

This study showed that pea protein isolate content in the feed formulation was the dominant factor governing the physical properties and aroma profile of high-protein corn-based extrudates, exceeding the influence of extrusion parameters such as screw speed and barrel temperature profile. Protein incorporation resulted in marked differences in physical properties compared to corn-only extrudates. However, no substantial differences were observed between formulations containing 30% and 50% protein, whereas incorporation at 70% protein led to pronounced changes. Acrylamide formation peaked at 30% protein and decreased at both 50% and 70% protein levels. Extrusion under the applied conditions did not promote acrylamide formation or lipid oxidation, confirming the suitability of pea protein isolate from a food safety point of view. Extrusion enhanced pyrazine formation, contributing desirable roasted and nutty aroma notes, without significantly affecting other aroma compounds. In terms of aroma profile, increasing the protein content to 70% shifted the samples to a distinct region in PCA space, clearly separated from the control. Considering all measured physical, chemical, and aroma-related parameters, the formulation containing 50% pea protein represents the most balanced option while achieving a high level of protein enrichment. Overall, these findings provide guidance for optimizing protein level and processing conditions in the development of high-protein extruded foods, taking texture, aroma, and safety into account. The specific mechanical energy values reported may serve as a reference for scale-up purposes. However, further optimization of processing conditions may be required to achieve these values at larger scale or may be a modelling approach is required, while accounting for the roles of formulation composition and processing parameters on extrudate properties as demonstrated in this study.

## CRediT authorship contribution statement

**Neslihan Göncüoğlu Taş:** Writing – original draft, Visualization, Methodology, Conceptualization. **Dimitris P. Balagiannis:** Writing – review & editing, Methodology. **Sameer Khalil Ghawi:** Writing – review & editing, Methodology. **Vural Gökmen:** Writing – review & editing, Conceptualization. **Jane K. Parker:** Writing – review & editing, Conceptualization.

## Declaration of competing interest

The authors declare the following financial interests/personal relationships which may be considered as potential competing interests: Given their role as VSI Guest Editor, Jane K Parker had no involvement in the peer-review of this article and has no access to information regarding its peer-review.

## Data Availability

Data will be made available on request.
