# Supplementary material for: Effect of extrusion parameters and feed composition on physical characteristics, aroma profile and acrylamide content in pea protein-enriched corn extrudates
Source: Food Chem X. 2026 May 22;36:104019. doi: 10.1016/j.fochx.2026.104019 (PMC13226939; doi:10.1016/j.fochx.2026.104019)
Supplement: Supplementary file 1 — Supplementary material [file mmc1.docx]

**Supplementary Material**

**Effect of extrusion parameters and feed composition on physical characteristics, aroma profile and acrylamide content in pea protein-enriched corn extrudates**

Neslihan Göncüoğlu Taş^1^, Dimitris P. Balagiannis^2^, Sameer Khalil Ghawi^2^, Vural Gökmen^1^, Jane K. Parker^2*^

^1^Food Quality and Safety (FoQuS) Research Group, Department of Food Engineering, Hacettepe University, 06800 Beytepe, Ankara, Turkiye

^2^Department of Food and Nutritional Sciences, University of Reading, Whiteknights, Reading RG6 6DZ, UK

**Table S1.** Linear Retention Indices (LRI) of aroma compounds and their quantification ions

| **RT, min** | **LRI calculated** | **LRI authentic** | **Compound name** | **Quantification ion** | **Source** |
| --- | --- | --- | --- | --- | --- |
| 3.958 | 553 | 557 | 2-methylpropanal | 72 | authentic |
| 4.537 | 586 | 600 | 2,3-butanedione | 86 | authentic |
| 4.635 | 592 | 600 | butanal | 72 | authentic |
| 4.740 | 598 | 604 | 2-butanone | 72 | authentic |
| 6.317 | 652 | 657 | 3-methylbutanal | 58 | authentic |
| 6.675 | 664 | 664 | 2-methylbutanal | 57 | authentic |
| 7.168 | 680 | 672 | 1-penten-3-ol | 57 | authentic |
| 7.591 | 694 | 696 | 2,3-pentadione | 100 | authentic |
| 7.689 | 698 | 701 | pentanal | 58 | authentic |
| 7.873 | 703 | 701 | 2-ethylfuran | 81 | authentic |
| 9.122 | 737 | 732 | 3-methylbutanol | 55 | authentic |
| 9.223 | 739 | 735 | pyrazine | 80 | authentic |
| 9.264 | 740 | 744 | 2-methylbutanol | 57 | authentic |
| 9.726 | 753 | 746 | dimethyldisulphide | 94 | authentic |
| 10.285 | 768 | 763 | 1-pentanol | 70 | authentic |
| 10.716 | 779 | 775 | butanoic acid | 60 | authentic |
| 11.120 | 790 | 791 | 2-hexanone | 58 | authentic |
| 11.515 | 801 | 802 | hexanal | 56 | authentic |
| 12.394 | 823 | 827 | 2-methylpyrazine | 94 | authentic |
| 12.604 | 829 | 839 | 3-methylbutanoic acid | 60 | authentic |
| 12.700 | 831 | 845 | 2-methylbutanoic acid | 74 | authentic |
| 12.805 | 834 | 836 | 2-furfural | 96 | authentic |
| 13.542 | 853 | 855 | (E)-2-hexenal | 83 | authentic |
| 13.600 | 854 | 855 | 2-furanmethanol | 98 | authentic |
| 14.093 | 867 | 867 | 1-hexanol | 56 | authentic |
| 14.323 | 873 | 897 | pentanoic acid | 60 | authentic |
| 15.000 | 890 | 893 | 2-heptanone | 71 | authentic |
| 15.446 | 902 | 903 | heptanal | 70 | authentic |
| 15.879 | 912 | 916 | 2,5(6)-dimethylpyrazine | 108 | authentic |
| 16.069 | 917 | 920 | 2-ethylpyrazine | 107 | authentic |
| 16.227 | 921 | 922 | 2,3-dimethylpyrazine | 67 | authentic |
| 17.726 | 958 | 959 | (E)-2-heptenal | 83 | authentic |
| 18.073 | 966 | 969 | benzaldehyde | 106 | authentic |
| 18.168 | 969 | 968 | 1-heptanol | 70 | authentic |
| 18.418 | 975 | 984 | hexanoic acid | 60 | authentic |
| 18.592 | 979 | 983 | 1-octen-3-ol | 57 | authentic |
| 18.807 | 985 | 985 | 2,3-octanedione | 99 | authentic |
| 18.921 | 987 | 987 | 6-methyl-5-hepten-2-one | 69 | authentic |
| 19.095 | 992 | 991 | 2-octanone | 58 | authentic |
| 19.177 | 994 | 992 | 2-pentylfuran | 81 | authentic |
| 19.465 | 1001 | 1002 | 2-ethyl-5-methylpyrazine | 121 | authentic |
| 19.508 | 1002 | 1001 | 2,3-dimethyl-2-cyclopenten-1-one | 110 | a |
| 19.585 | 1004 | 1002 | 2-ethyl-6-methylpyrazine | 121 | authentic |
| 19.587 | 1004 | 1007 | octanal | 84 | authentic |
| 19.629 | 1005 | 1008 | trimethylpyrazine | 122 | authentic |
| 19.737 | 1008 | 1006 | 2-ethyl-3-methylpyrazine | 121 | authentic |
| 20.246 | 1020 | 1020 | 2-ethenyl-6-methylpyrazine | 120 | b |
| 21.049 | 1041 | 1040 | (E)-3-octen-2-one | 111 | authentic |
| 21.444 | 1050 | 1058 | phenylacetaldehyde | 91 | authentic |
| 21.855 | 1061 | 1063 | E-2-octenal | 83 | authentic |
| 22.238 | 1070 | 1073 | 1-octanol | 56 | authentic |
| 22.361 | 1073 | 1071 | (E,E)-3,5-octadien-2-one | 95 | c |
| 22.734 | 1083 | 1087 | 3-ethyl-2,5-dimethylpyrazine | 135 | authentic |
| 23.145 | 1093 | 1092 | 2-nonanone | 58 | authentic |
| 23.256 | 1096 | 1096 | (E,Z)-3,5-octadien-2-one | 95 | d |
| 23.604 | 1105 | 1107 | 2-methyl-6-(1-propenyl)pyrazine | 133 | e |
| 23.657 | 1106 | 1107 | nonanal | 57 | authentic |
| 25.709 | 1160 | 1157 | 2,3-diethyl-5(or 6)-methylpyrazine | 149 | authentic |
| 25.829 | 1163 | 1157 | 2-methyl-3,5-diethylpyrazine | 149 | f |
| 25.952 | 1167 | 1162 | 2,5-dimethyl-3-propylpyrazine | 122 | g |
| 26.100 | 1171 | 1176 | 1-nonanol | 70 | authentic |
| 27.015 | 1195 | 1193 | 2-decanone | 58 | h |
| 31.445 | 1318 | 1333 | (E,E)-2.4-decadienal | 81 | authentic |

*a: Lu et. al., 1997; b and f: van Loon et. al., 2005; c: Radulovic et. al., 2010; d: Methven et. al., 2007; e: Risticevic et. al., 2007; g: Bolyston and Viniyard, 1998; h: Elmore et. al., 2000

**Table S2.** Free amino acid and sugar profile of pea protein isolate and cornmeal

|  | **Pea Protein Isolate (PPI)** | **Cornmeal (CM)** |
| --- | --- | --- |
| *Amino acids (mg/kg)* | | |
| Ala | 57±9 | 38±10 |
| Arg | 119±11 | 33±4* |
| Asn | 15±5 | 128±6* |
| Asp | 24±9 | 245±21* |
| Gln | 18±4 | 10±1* |
| Glu | 13±3 | 81±17* |
| Gly | 19±4 | 18±4 |
| His | 30±9 | 32±19* |
| Hyp | 174±32 | 27±6* |
| Ile | 167±36 | 24±4* |
| Leu | 211±35 | 9±1* |
| Lys | 150±48 | 27±6* |
| Met | 157±74 | 16±3* |
| Phe | 145±22 | 14±1* |
| Pro | 12±2 | 101±30 |
| Ser | 25±4 | 19±5 |
| Thr | 28±18 | 10±3 |
| Trp | 32±2 | 10±4* |
| Tyr | 450±539 | 496±189 |
| Val | 57±9 | 15±2* |
| Total | 1904±876 | 1355±335 |
| *Sugars (%)* | | |
| Fru | 0.01±0.003 | 0.2±0.001* |
| Glc | 0.001±0.001 | 0.2±0.002* |
| Mal | nd | nd |
| Suc | 0.02±0.01 | 0.6±0.03* |
| Total | 0.031±0.014 | 1.0±0.033* |

*indicates siginificant difference (*p<0.05*) according to t-test between samples.

*nd: not detected.

**Table S3.** Aroma compounds of cornmeal-pea protein isolate extrudates with different compositions at varying moisture contents and their raw materials (µg/kg)

|  |  | CM | PPI | E1 | E2 | E3 | E4 | E5 | E6 | E7 | E8 | E9 |
| --- | --- | --- | --- | --- | --- | --- | --- | --- | --- | --- | --- | --- |
|  |  |  |  | CM:PPI  (100:0)-M15 | CM:PPI (85:15)-M15 | CM:PPI  (70:30)-M15 | CM:PPI  (50:50)-M17 | CM:PPI (50:50)-M16 | CM:PPI  (50:50)-M15 | CM:PPI  (30:70)-M17 | CM:PPI  (30:70)-M16 | CM:PPI  (30:70)-M15 |
|  | Strecker aldehydes |  |  |  |  |  |  |  |  |  |  |  |
| 2-MP | 2-methylpropanal | nd | nd | 28±2^d^ | 73±41^bcd^ | 120±11^ab^ | 104±6^abc^ | 127±16^a^ | 155±8^a^ | 42±7^d^ | 56±13^cd^ | 68±15^bcd^ |
| 3-MBL | 3-methylbutanal | 27±1^g^ | 222±40^efg^ | 193±20^fg^ | 540±271^cde^ | 1065±82^ab^ | 903±121^b^ | 1058±84^ab^ | 1249±42^a^ | 507±133^def^ | 776±54^bcd^ | 871±160^bc^ |
| 2-MBL | 2-methylbutanal | 11±0^f^ | nd | 222±11^ef^ | 404±209^cde^ | 735±81^ab^ | 607±78^abc^ | 703±43^ab^ | 844±31^a^ | 310±70^de^ | 483±30^bcd^ | 548±66^bcd^ |
| PAL | phenylacetaldehyde | 90±24^c^ | 713±193^bc^ | 2424±1038^ab^ | 2327±848^abc^ | 2119±221^abc^ | 2063±272^abc^ | 1873±189^abc^ | 2126±519^abc^ | 2647±858^ab^ | 4080±939^a^ | 2938±1716^ab^ |
|  | Lipid-derived aldehydes |  |  |  |  |  |  |  |  |  |  |  |
| BL | butanal | nd | 442±75^abc^ | nd | 109±59^c^ | 276±25^bc^ | 366±41^abc^ | 374±34^abc^ | 430±29^abc^ | 337±92^abc^ | 630±305^a^ | 454±63^ab^ |
| PL | pentanal | 90±5^e^ | 4840±847^a^ | 210±50^e^ | 775±371^de^ | 1706±165^cd^ | 2251±226^bc^ | 2405±81^bc^ | 2701±144^bc^ | 2783±856^bc^ | 3383±360^b^ | 3172±616^b^ |
| HXL | hexanal | 11796±2343^d^ | 470318±95871^ab^ | 6797±1895^d^ | 67878±31769^d^ | 177830±20179^d^ | 173071±22214^d^ | 183833±6210^cd^ | 205595±18203^bcd^ | 453181±170602^ab^ | 564558±53734^a^ | 445738±220388^abc^ |
| 2-HXL | (E)-2-hexenal | nd | 1959±411^a^ | nd | 161±67^c^ | 221±30^c^ | 527±50^bc^ | 549±17^bc^ | 629±134^bc^ | 1394±539^ab^ | 1557±246^a^ | 1117±470^ab^ |
| HPL | heptanal | 460±97^e^ | 5393±1408^bcde^ | 298±94^e^ | 1634±691^de^ | 4174±521^cde^ | 5916±934^bcde^ | 6365±298^bcde^ | 7412±1065^abcd^ | 9039±3925^abc^ | 13069±1714^a^ | 10716±5149^ab^ |
| 2-HPL | (E)-2-heptenal | 266±58^de^ | 2819±643^a^ | 166±67^e^ | 267±98^de^ | 242±32^de^ | 682±88^cde^ | 1003±40^bcde^ | 1213±267^bcde^ | 1567±643^bc^ | 1863±392^ab^ | 1258±636^bcd^ |
| OCL | octanal | 262±58^de^ | 2913±815^ab^ | 134±73^e^ | 532±223^cde^ | 1902±240^abcde^ | 1790±322^bcde^ | 1911±150^abcde^ | 2333±412^abcd^ | 2602±1189^abc^ | 3915±610^a^ | 3154±1649^ab^ |
| E-2-OCL | (E)-2-octenal | 318±80^d^ | 2089±482^a^ | 66±28^d^ | 261±106^d^ | 514±79^cd^ | 812±103^bcd^ | 816±51^bcd^ | 935±217^bcd^ | 1694±612^ab^ | 2212±337^a^ | 1494±730^abc^ |
| NL | nonanal | 2604±533^bc^ | 13558±3665^a^ | 1754±1051^c^ | 2646±1198^bc^ | 8229±867^abc^ | 8481±1522^abc^ | 7959±720^abc^ | 9539±1144^ab^ | 9606±4085^ab^ | 13659±2004^a^ | 10778±5450^a^ |
| 2,4-DD | (E,E)-2,4-decadienal | 74±9^c^ | 988±303^ab^ | 93±41^c^ | 352±130^bc^ | 308±55^bc^ | 648±128^bc^ | 536±107^bc^ | 470±137^bc^ | 994±402^ab^ | 1473±427^a^ | 801±553^abc^ |
|  | Other aldehydes |  |  |  |  |  |  |  |  |  |  |  |
| BZL | benzaldehyde | 169±56^d^ | 35554±8405^a^ | 1677±711^d^ | 6496±2587^cd^ | 14655±2019^bcd^ | 13339±1744^bcd^ | 13486±867^bcd^ | 15811±3565^bcd^ | 19631±8279^bc^ | 27582±6136^ab^ | 21508±10867^abc^ |
| FL | 2-furfural | nd | 242±42^f^ | 413±44^ef^ | 1084±593^bcd^ | 1743±124^ab^ | 1544±180^abc^ | 1591±212^abc^ | 1918±134^a^ | 506±126^def^ | 749±165^def^ | 973±40^cde^ |
|  | Diketones |  |  |  |  |  |  |  |  |  |  |  |
| 2,3-BD | 2,3-butanedione | nd | nd | 99±47^ab^ | 120±57^a^ | 105±5^ab^ | 104±1^ab^ | 99±5^ab^ | 116±14^a^ | 33±5^b^ | 47±6^ab^ | 59±6^ab^ |
| 2,3-PD | 2,3-pentanedione | nd | 117±15^ab^ | 45±15^b^ | 137±73^a^ | 93±1^ab^ | 89±7^ab^ | 82±8^ab^ | 101±10^ab^ | nd | nd | nd |
| 2,3-OD | 2,3-octanedione | 69±13^d^ | 3127±753^ab^ | 24±9^d^ | 518±212^d^ | 1245±157^cd^ | 1466±215^bcd^ | 1533±107^bcd^ | 1830±321^abcd^ | 2459±1103^abc^ | 3535±607^a^ | 2700±1431^abc^ |
|  | Ketones |  |  |  |  |  |  |  |  |  |  |  |
| BO | 2-butanone | nd | 1417±204^a^ | 39±8^c^ | 242±122^c^ | 628±71^b^ | 655±75^b^ | 654±45^b^ | 801±67^b^ | 571±145^b^ | 702±70^b^ | 789±97^bcd^ |
| 2-HXO | 2-hexanone | nd | 855±126^cd^ | nd | 253±114^d^ | 744±73^cd^ | 914±87^cd^ | 991±16^cd^ | 1221±119^bc^ | 1564±512^abc^ | 2163±247^a^ | 1954±713^b^ |
| 2-HPO | 2-heptanone | 172±37^d^ | 8570±1889^cd^ | 94±26^d^ | 3354±1492^d^ | 14532±2159^cd^ | 20163±3233^cd^ | 23265±1501^bcd^ | 29497±3624^abc^ | 32959±14410^abc^ | 52991±6285^a^ | 47481±22965^ab^ |
| 6-M-5-HPO | 6-methyl-5-hepten-2-one | 301±100^a^ | 358±110^a^ | 320±134^a^ | 281±131^a^ | 425±39^a^ | 278±28^a^ | 306±33^a^ | 357±110^a^ | 465±200^a^ | 656±93^a^ | 533±302^a^ |
| 2-OCO | 2-octanone | nd | 6186±1728^ab^ | 158±79^d^ | 1123±446^cd^ | 3601±565^bcd^ | 3594±558^bcd^ | 3969±329^abcd^ | 5044±1075^abc^ | 5373±2484^abc^ | 8211±1297^a^ | 7014±3309^abc^ |
| CYCPO | 2,3-dimethyl-2-cyclopenten-1-one | nd | 511±142^cd^ | nd | 132±56^d^ | 383±52^cd^ | 574±83^cd^ | 559±46^cd^ | 720±168^bcd^ | 1243±501^abc^ | 1794±323^a^ | 1524±681^ab^ |
| E-3-OC-2-O | E-3-octen-2-one | nd | 2027±592^a^ | 222±63^d^ | 521±208^cd^ | 799±88^bcd^ | 835±116^bcd^ | 789±84^bcd^ | 878±172^bcd^ | 1353±544^abc^ | 1813±340^ab^ | 1226±774^abcd^ |
| E,E-3,5-OCDO | (E,E)-3,5-octadien-2-one | nd | 2135±613^a^ | nd | 122±54^b^ | 271±60^b^ | 343±53^b^ | 332±49^b^ | 386±84^b^ | 558±267^b^ | 848±204^b^ | 594±326^b^ |
| 2-NO | 2-nonanone | nd | 5066±1522^bcd^ | nd | 745±316^d^ | 3174±542^cd^ | 6380±1141^abcd^ | 6944±694^abcd^ | 9168±2029^abc^ | 8635±4150^abcd^ | 13706±2263^a^ | 11622±6078^ab^ |
| E,Z-3,5-OCDO | (E,Z)-3,5-octadien-2-one | nd | 16917±4092^a^ | nd | 1098±414^c^ | 2875±489^bc^ | 3294±488^bc^ | 3291±398^bc^ | 3807±920^bc^ | 5953±2670^bc^ | 8981±1998^b^ | 6305±3396^bc^ |
| 2-DO | 2-decanone | nd | 3332±924^bcd^ | nd | 444±191^d^ | 2123±407^cd^ | 4499±774^abcd^ | 4748±513^abcd^ | 6432±1555^abc^ | 5712±2599^abcd^ | 9316±1754^a^ | 7837±4097^ab^ |
|  | Alcohols |  |  |  |  |  |  |  |  |  |  |  |
| 1-P-3OL | 1-penten-3-ol | 101±19^de^ | 3010±413^a^ | 44±19^e^ | 382±180^cde^ | 892±68^b^ | 619±49^bc^ | 624±31^bc^ | 714±70^bc^ | 523±119^bcd^ | 637±83^bc^ | 679±21^bc^ |
| 3-MBOL | 3-methylbutanol | 131±22^a^ | nd | 100±50^a^ | nd | nd | nd | nd | nd | nd | nd | nd |
| 2-MBOL | 2-methylbutanol | 53±10^a^ | nd | 46±22^a^ | nd | nd | nd | nd | nd | nd | nd | nd |
| 1-POL | 1-pentanol | 515±120^b^ | 4480±736^a^ | 224±113^b^ | 523±243^b^ | 1684±116^b^ | 1436±59^b^ | 1393±23^b^ | 1592±188^b^ | 3429±924^a^ | 4040±605^a^ | 3524±1307^a^ |
| 1-HXOL | 1-hexanol | 4472±1281^bc^ | 7655±1591^ab^ | 1181±546^c^ | 1785±764^c^ | 5047±594^bc^ | 3941±408^bc^ | 3863±46^bc^ | 4615±852^bc^ | 8226±2844^ab^ | 10410±1919^a^ | 8679±3545^ab^ |
| 1-HPOL | 1-heptanol | 713±169^bcd^ | 1489±296^ab^ | 83±44^d^ | 128±75^d^ | 568±80^cd^ | 498±72^cd^ | 523±55^cd^ | 624±168^bcd^ | 1245±508^abc^ | 1743±363^a^ | 1330±695^abc^ |
| 1-O-3-OL | 1-octen-3-ol | 2036±590^de^ | 16397±3284^a^ | 548±234^e^ | 1545±654^de^ | 3708±564^cde^ | 5658±745^cde^ | 7315±350^bcde^ | 8726±1873^abcd^ | 10510±4960^abc^ | 14923±3108^ab^ | 11254±5660^abc^ |
| 1-OCOL | 1-octanol | 524±142^de^ | 2095±544^ab^ | 49±26^e^ | 132±62^e^ | 535±65^de^ | 739±94^cde^ | 751±119^cde^ | 1014±257^bcde^ | 1600±705^abcd^ | 2242±526^a^ | 1795±869^abc^ |
| 1-NOL | 1-nonanol | nd | 601±134^a^ | nd | 28±13^c^ | 110±10^bc^ | 137±26^bc^ | 133±16^bc^ | 177±42^bc^ | 364±131^ab^ | 503±84^a^ | 365±174^ab^ |
|  | Furans |  |  |  |  |  |  |  |  |  |  |  |
| EF | 2-ethylfuran | nd | 958±281^cde^ | nd | 567±368^e^ | 1511±154^abcde^ | 1952±391^abc^ | 2049±395^ab^ | 2169±434^a^ | 947±315^de^ | 1117±256^bcde^ | 1608±342^abcd^ |
| PF | 2-pentylfuran | 6702±656^d^ | 236548±66730^a^ | 1097±728^d^ | 27556±17291^cd^ | 123039±14606^b^ | 89632±20136^bc^ | 90448±15286^bc^ | 108489±1974^b^ | 58990±20254^bcd^ | 80928±14937^bc^ | 102848±12784^b^ |
|  | Sulfur compounds |  |  |  |  |  |  |  |  |  |  |  |
| DMDS | dimethyl disulfide | 46±3^d^ | 228±148^abcd^ | 56±32^cd^ | 105±61^bcd^ | 77±23^cd^ | 465±139^abc^ | 568±363^a^ | 373±76^abcd^ | 504±117^ab^ | 535±98^a^ | 521±140^ab^ |
|  | Acids |  |  |  |  |  |  |  |  |  |  |  |
| BA | butanoic acid | nd | 3775±81^abc^ | nd | 208±106^c^ | 394±16^c^ | 1353±189^bc^ | 1270±178^bc^ | 1620±540^bc^ | 3994±2292^abc^ | 6354±2702^a^ | 4965±2542^ab^ |
| 3-MBA | 3-methylbutanoic acid | nd | 2472±166^ab^ | nd | 153±69^c^ | 298±16^c^ | 924±152^bc^ | 825±83^bc^ | 1110±390^bc^ | 2187±1162^abc^ | 3344±1252^a^ | 2609±1246^ab^ |
| 2-MBA | 2-methylbutanoic acid | nd | 665±24^ab^ | nd | 42±18^c^ | 70±9^c^ | 238±18^bc^ | 214±10^bc^ | 295±100^abc^ | 525±297^abc^ | 830±327^a^ | 655±300^ab^ |
| PA | pentanoic acid | nd | 8360±627^ab^ | nd | 263±159^b^ | 697±78^b^ | 2504±419^b^ | 2429±507^b^ | 3347±1451^ab^ | 6661±4211^ab^ | 11192±5253^a^ | 8140±4967^ab^ |
| HXA | hexanoic acid | 172±99^c^ | 37224±5551^ab^ | 79±22^c^ | 703±501^bc^ | 2530±229^bc^ | 10854±2002^bc^ | 11089±2862^bc^ | 16177±8288^abc^ | 26120±18486^abc^ | 51803±27054^a^ | 34624±24159^abc^ |
|  | Pyrazines |  |  |  |  |  |  |  |  |  |  |  |
| P1 | pyrazine | nd | nd | nd | 12±12^a^ | 17±1^a^ | 7±1^a^ | 8±3^a^ | 10±1^a^ | nd | nd | nd |
| P2 | 2-methylpyrazine | nd | nd | 16±7^c^ | 175±157^abc^ | 295±15^a^ | 142±24^abc^ | 154±44^abc^ | 208±28^ab^ | 45±14^bc^ | 62±15^bc^ | 85±10^bc^ |
| P3 | 2,5(6)-dimethylpyrazine | nd | nd | 103±47^b^ | 1794±1611^ab^ | 2909±225^a^ | 1137±213^b^ | 1270±325^ab^ | 1720±242^ab^ | 200±81^b^ | 377±103^b^ | 528±75^b^ |
| P4 | 2-ethylpyrazine | nd | nd | 3±3^b^ | 14±14^b^ | 90±59^a^ | 13±2^b^ | 15±4^b^ | 18±2^b^ | 4±1^b^ | 5±1^b^ | 8±1^b^ |
| P5 | 2,3-dimethylpyrazine | nd | nd | 1±0^c^ | 17±15^abc^ | 32±3^a^ | 15±3^bc^ | 16±4^abc^ | 21±3^ab^ | 5±1^bc^ | 7±2^bc^ | 9±1^bc^ |
| P6 | 2-ethyl-5-methylpyrazine | nd | nd | 2±1^c^ | 38±34^abc^ | 66±5^a^ | 30±6^abc^ | 34±8^abc^ | 43±5^ab^ | 9±4^bc^ | 15±3^bc^ | 17±2^bc^ |
| P7 | 2-ethyl-6-methylpyrazine | nd | nd | 2±1^b^ | 107±96^ab^ | 65±6^ab^ | 91±21^ab^ | 105±24^ab^ | 156±22^a^ | 22±9^b^ | 39±11^b^ | 43±25^b^ |
| P8 | trimethylpyrazine | nd | nd | 7±3^d^ | 195±174^bcd^ | 512±55^a^ | 177±37^bcd^ | 222±52^bc^ | 333±42^ab^ | 33±15^cd^ | 65±16^cd^ | 94±14^cd^ |
| P9 | 2-ethyl-3-methylpyrazine | nd | nd | nd | 11±10^ab^ | 25±1^ab^ | 12±2^ab^ | 14±3^ab^ | 17±2^ab^ | 4±1^b^ | 6±2^ab^ | 35±25^a^ |
| P10 | 2-ethenyl-6-methylpyrazine | nd | nd | 5±2^b^ | 95±83^a^ | 80±6^ab^ | 52±12^ab^ | 47±10^ab^ | 43±6^ab^ | 4±2^b^ | 10±3^ab^ | 9±2^ab^ |
| P11 | 3-ethyl-2,5-dimethylpyrazine | nd | nd | 28±10^b^ | 1115±1011^ab^ | 1827±200^a^ | 653±144^b^ | 756±166^ab^ | 960±137^ab^ | 86±37^b^ | 195±53^b^ | 262±50^b^ |
| P12 | 2-methyl-6-1-propenylpyrazine | nd | nd | nd | 23±20^abc^ | 40±3^a^ | 20±4^abc^ | 22±5^abc^ | 28±4^ab^ | 3±2^c^ | 7±2^bc^ | 8±3^bc^ |
| P13 | 2,3-diethyl-5(or 6)-methylpyrazine | nd | nd | nd | 3±3^a^ | 6±1^a^ | 2±0^a^ | 2±1^a^ | 4±0^a^ | nd | nd | nd |
| P14 | 2-methyl-3,5-diethylpyrazine | nd | nd | nd | 15±13^bc^ | 32±4^a^ | 12±3^bc^ | 14±3^bc^ | 20±3^ab^ | 1±1^c^ | 3±1^bc^ | 5±1^bc^ |
| P15 | 3-propyl-2,5-dimethylpyrazine | nd | nd | nd | 34±30^bc^ | 80±8^a^ | 32±8^bc^ | 35±7^bc^ | 44±8^ab^ | 5±2^c^ | 10±3^bc^ | 12±3^bc^ |

*The values within the same row followed by the same letters are not significantly different (*p =* 0.05) according to Tukey’s test.

**nd: not detected.

**Table S4.** Aroma compounds of cornmeal–pea protein isolate extrudates (50:50 and 30:70 blends) at varying screw speeds and barrel temperature profiles (µg/kg)

|  |  | E10 | E11 | E6 | E12 | E13 | E14 | E15 | E16 | E9 | E17 | E18 | E19 |
| --- | --- | --- | --- | --- | --- | --- | --- | --- | --- | --- | --- | --- | --- |
|  |  | CM:PPI  (50:50)-SS500 | CM:PPI  (50:50)-SS450 | CM:PPI  (50:50)-SS400 | CM:PPI  (50:50)-T2 | CM:PPI  (50:50)-T3 | CM:PPI  (50:50)-T4 | CM:PPI  (30:70)-SS500 | CM:PPI  (30:70)-SS450 | CM:PPI  (30:70)-SS400 | CM:PPI  (30:70)-T2 | CM:PPI  (30:70)-T3 | CM:PPI  (30:70)-T4 |
|  | Strecker aldehydes |  |  |  |  |  |  |  |  |  |  |  |  |
| 2-MP | 2-methylpropanal | 158±11^a^ | 156±7^a^ | 155±8^a^ | 145±38^a^ | 150±20^a^ | 189±7^a^ | 132±22^ab^ | 75±8^b^ | 68±15^b^ | 167±14^a^ | 184±45^a^ | 192±33^a^ |
| 3-MBL | 3-methylbutanal | 1361±3^abc^ | 1295±68^abc^ | 1249±42^bc^ | 1234±318^bc^ | 1356±166^abc^ | 1631±43^ab^ | 1231±417^bc^ | 1089±133^bc^ | 871±160^c^ | 1547±144^ab^ | 1703±353^ab^ | 1912±311^a^ |
| 2-MBL | 2-methylbutanal | 994±44^abc^ | 915±46^abcd^ | 844±31^abcd^ | 860±221^abcd^ | 996±111^abc^ | 1174±45^ab^ | 771±280^bcd^ | 668±54^cd^ | 548±66^d^ | 994±89^abc^ | 1134±165^ab^ | 1243±201^a^ |
| PAL | phenylacetaldehyde | 1601±175^b^ | 1764±343^b^ | 2126±519^b^ | 1521±508^b^ | 1600±115^b^ | 1678±238^b^ | 1670±509^b^ | 4835±2168^a^ | 2938±1716^ab^ | 1638±220^b^ | 1748±542^b^ | 1925±592^b^ |
|  | Lipid-derived aldehydes |  |  |  |  |  |  |  |  |  |  |  |  |
| BL | butanal | 384±24^cde^ | 364±33^c^ | 430±29^c^ | 350±101^c^ | 331±30^c^ | 360±16^c^ | 701±155^ab^ | 469±38^bc^ | 454±63^bc^ | 711±69^ab^ | 746±167^a^ | 744±158^a^ |
| PL | pentanal | 2406±152^b^ | 2229±194^b^ | 2701±144^b^ | 2214±614^b^ | 2032±180^b^ | 2249±85^b^ | 3888±1125^ab^ | 3662±429^b^ | 3172±616^ab^ | 4725±482^a^ | 4709±1069^a^ | 4759±1131^a^ |
| HXL | hexanal | 174468±11254^b^ | 160853±17235^b^ | 205595±18203^b^ | 177916±53382^b^ | 165355±14421^b^ | 176994±14267^b^ | 166182±70386^b^ | 566178±19451^a^ | 445738±220388^a^ | 221797±24203^b^ | 213154±48537^b^ | 214224±59672^b^ |
| 2-HXL | (E)-2-hexenal | 497±36^c^ | 408±71^c^ | 629±134^bc^ | 431±151^c^ | 276±138^c^ | 443±49^c^ | 452±196^c^ | 1490±293^a^ | 1117±470^ab^ | 594±112^bc^ | 530±144^bc^ | 567±194^bc^ |
| HPL | heptanal | 6302±591^bc^ | 5921±966^bc^ | 7412±1065^bc^ | 6317±2106^bc^ | 6500±492^bc^ | 6767±944^bc^ | 4554±2342^c^ | 14843±2182^a^ | 10716±5149^ab^ | 6033±652^bc^ | 5750±1559^bc^ | 6023±1787^bc^ |
| 2-HPL | (E/Z)-2-heptenal | 948±101^b^ | 592±105^b^ | 1213±267^ab^ | 833±301^b^ | 522±42^b^ | 893±123^b^ | 497±220^b^ | 1982±453^a^ | 1258±636^ab^ | 644±75^b^ | 529±154^b^ | 787±341^b^ |
| OCL | octanal | 1917±187^b^ | 1735±331^b^ | 2333±412^b^ | 1901±654^b^ | 1903±151^b^ | 2131±269^b^ | 1397±704^b^ | 4899±1140^a^ | 3154±1649^ab^ | 1772±195^b^ | 1627±479^b^ | 1712±511^b^ |
| E-2-OCL | (E)-2-octenal | 717±73^b^ | 635±93^b^ | 935±217^b^ | 668±238^b^ | 591±52^b^ | 698±73^b^ | 564±125^b^ | 2363±933^a^ | 1494±730^ab^ | 964±99^b^ | 839±235^b^ | 838±257^b^ |
| NL | nonanal | 7605±1174^b^ | 7469±1591^b^ | 9539±1144^b^ | 7173±2238^b^ | 7987±1728^b^ | 9132±303^b^ | 5973±2397^b^ | 18324±5606^a^ | 10778±5450^ab^ | 6795±1306^b^ | 2845±3439^b^ | 5838±1296^b^ |
| 2.4-DD | (E,E)-2,4-decadienal | 270±60^bc^ | 263±67^bc^ | 470±137^abc^ | 242±116^bc^ | 203±25^bc^ | 179±31^c^ | 397±341^bc^ | 1087±219^a^ | 801±553^ab^ | 482±58^abc^ | 329±110^bc^ | 284±84^bc^ |
|  | Other aldehydes |  |  |  |  |  |  |  |  |  |  |  |  |
| BZL | benzaldehyde | 12228±1183^b^ | 11952±2137^b^ | 15811±3565^b^ | 12371±4516^b^ | 12406±963^b^ | 13442±1822^b^ | 12595±4279^b^ | 36778±17848^a^ | 21508±10867^ab^ | 12947±1607^b^ | 12514±3757^b^ | 13279±4387^b^ |
| FL | 2-furfural | 2147±83^ab^ | 2031±110^abc^ | 1918±134^abc^ | 1805±553^bcd^ | 2075±316^abc^ | 2777±126^a^ | 1245±94^cd^ | 1004±134^d^ | 973±40^d^ | 1801±485^bcd^ | 2145±474^ab^ | 2449±372^ab^ |
|  | Diketones |  |  |  |  |  |  |  |  |  |  |  |  |
| 2.3-BD | 2,3-butanedione | 100±5^cde^ | 102±17^cde^ | 116±14^abcd^ | 87±26^cde^ | 86±13^cde^ | 106±4^bcd^ | 127±15^abc^ | 40±5^e^ | 59±6^de^ | 141±16^abc^ | 166±44^ab^ | 172±40^a^ |
| 2.3-PD | 2,3-pentanedione | 76±5^ab^ | 86±13^a^ | 101±10^a^ | 61±14^b^ | 61±5^b^ | 68±1^ab^ | nd | nd | nd | 75±14^ab^ | 79±12^ab^ | 88±20^ab^ |
| 2.3-OD | 2,3-octanedione | 1383±155^b^ | 1347±230^b^ | 1830±321^b^ | 1369±508^b^ | 1363±101^b^ | 1375±209^b^ | 1180±612^b^ | 4061±1082^a^ | 2700±1431^ab^ | 1465±159^b^ | 1329±388^b^ | 1068±658^b^ |
|  | Ketones |  |  |  |  |  |  |  |  |  |  |  |  |
| BO | 2-butanone | 729±36^cde^ | 715±86^c^ | 801±67^c^ | 682±182^c^ | 686±74^c^ | 835±21^bc^ | 1443±290^ab^ | 831±133^bc^ | 789±97^c^ | 1661±172^a^ | 1871±455^a^ | 1958±389^a^ |
| 2-HXO | 2-hexanone | 1216±95^a^ | 1099±95^a^ | 1221±119^a^ | 1124±348^a^ | 1195±139^a^ | 1327±85^a^ | 914±395^b^ | 2419±167^a^ | 1954±713^a^ | 9239±13921^a^ | 1363±283^a^ | 1406±309^a^ |
| 2-HPO | 2-heptanone | 27822±2189^b^ | 25979±3849^b^ | 29497±3624^b^ | 28192±9483^b^ | 31700±2925^b^ | 32412±3741^b^ | 21236±12122^b^ | 66306±4754^a^ | 47481±22965^ab^ | 29496±2799^b^ | 31264±8641^b^ | 34353±9752^b^ |
| 6-M-5-HPO | 6-methyl-5-hepten-2-one | 342±28^a^ | 246±57^a^ | 357±110^a^ | 323±113^a^ | 299±28^a^ | 385±45^a^ | 261±133^a^ | 796±143^a^ | 533±302^a^ | 305±20^a^ | 628±449^a^ | 584±454^a^ |
| 2-OCO | 2-octanone | 4332±515^b^ | 4035±870^b^ | 5044±1075^b^ | 4433±1634^b^ | 4855±385^b^ | 5325±965^b^ | 3186±1855^b^ | 10655±2661^a^ | 7014±3309^ab^ | 4288±430^b^ | 2556±2209^b^ | 4583±1498^b^ |
| CYCPO | 2,3-dimethyl-2-cyclopenten-1-one | 607±35^bc^ | 605±115^bc^ | 720±168^bc^ | 577±204^c^ | 590±59^c^ | 650±79^bc^ | 587±209^c^ | 2114±577^a^ | 1524±681^ab^ | 710±106^bc^ | 874±336^bc^ | 991±377^bc^ |
| E-3-OC-2-O | E-3-octen-2-one | 592±80^b^ | 598±103^b^ | 878±172^b^ | 625±226^b^ | 558±34^b^ | 546±85^b^ | 498±271^b^ | 1845±659^a^ | 1226±774^ab^ | 587±67^b^ | 484±150^b^ | 491±151^b^ |
| E.E-3.5-OCDO | (E,E)-3,5-octadien-2-one | 283±39^b^ | 278±76^b^ | 386±84^b^ | 267±106^b^ | 260±21^b^ | 264±60^b^ | 301±150^b^ | 931±299^a^ | 594±326^ab^ | 314±10^b^ | 326±80^b^ | 397±188^b^ |
| 2-NO | 2-nonanone | 7722±862^b^ | 7122±1490^b^ | 9168±2029^b^ | 7920±2906^b^ | 8464±608^b^ | 8551±1612^b^ | 5682±3237^b^ | 20113±7137^a^ | 11622±6078^ab^ | 7161±590^b^ | 6654±2113^b^ | 7145±2387^b^ |
| E.Z-3.5-OCDO | (E,Z)-3,5-octadien-2-one | 2783±296^b^ | 2770±600^b^ | 3807±920^b^ | 2810±1099^b^ | 2685±214^b^ | 2659±500^b^ | 3346±1701^b^ | 10167±3573^a^ | 6305±3396^ab^ | 3911±470^b^ | 3393±1030^b^ | 3560±1261^b^ |
| 2-DO | 2-decanone | 5327±580^b^ | 4966±1049^b^ | 6432±1555^b^ | 5306±1911^b^ | 5627±410^b^ | 5918±1031^b^ | 3852±2051^b^ | 14273±5962^a^ | 7837±4097^ab^ | 4730±386^b^ | 4323±1367^b^ | 4643±1545^b^ |
|  | Alcohols |  |  |  |  |  |  |  |  |  |  |  |  |
| 1-P-3OL | 1-penten-3-ol | 663±12^cd^ | 632±66^bcd^ | 714±70^bcd^ | 534±145^d^ | 513±66^d^ | 654±44^cd^ | 783±191^bcd^ | 658±42^cd^ | 679±21^cd^ | 1068±165^abc^ | 1131±299^ab^ | 1265±283^a^ |
| 1-POL | 1-pentanol | 1422±93^c^ | 1368±154^c^ | 1592±188^c^ | 1352±389^c^ | 1300±111^c^ | 1549±94^c^ | 1443±321^abcd^ | 4064±367^a^ | 3524±1307^ab^ | 1838±304^c^ | 1948±474^c^ | 2115±522^bc^ |
| 1-HXOL | 1-hexanol | 3984±229^c^ | 3826±627^c^ | 4615±852^bc^ | 3689±1210^c^ | 3680±254^c^ | 4074±448^c^ | 3821±1508^c^ | 10274±590^a^ | 8679±3545^ab^ | 5055±733^bc^ | 4308±1895^c^ | 5585±1690^bc^ |
| 1-HPOL | 1-heptanol | 503±56^b^ | 498±46^b^ | 624±168^ab^ | 515±176^b^ | 478±44^b^ | 553±69^b^ | 456±231^b^ | nd | 1330±695^a^ | 610±91^ab^ | 571±161^b^ | 614±201^ab^ |
| 1-O-3-OL | 1-octen-3-ol | 7151±732^b^ | 5592±1049^b^ | 8726±1873^ab^ | 6959±2688^b^ | 5824±448^b^ | 7924±1101^ab^ | 6543±4111^b^ | 16321±2030^a^ | 11254±5660^ab^ | 8779±2419^ab^ | 7938±2299^ab^ | 11322±4456^ab^ |
| 1-OCOL | 1-octanol | 844±64^b^ | 713±106^b^ | 1014±257^b^ | 818±288^b^ | 739±43^b^ | 939±61^b^ | 1527±1486^ab^ | 2764±703^a^ | 1795±869^ab^ | 1054±232^b^ | 1014±269^b^ | 1074±372^b^ |
| 1-NOL | 1-nonanol | 144±18^b^ | 130±36^b^ | 177±42^b^ | 148±62^b^ | 133±12^b^ | 171±17^b^ | 222±153^b^ | 597±193^a^ | 365±174^ab^ | 235±18^b^ | 226±54^b^ | 249±64^b^ |
|  | Furans |  |  |  |  |  |  |  |  |  |  |  |  |
| EF | 2-ethylfuran | 2776±302^b^ | 2345±260^b^ | 2169±434^b^ | 2387±581^b^ | 2929±706^b^ | 2309±317^b^ | 4201±2038^ab^ | 1386±416^a^ | 1608±342^b^ | 7747±3754^a^ | 7215±388^a^ | 5035±1896^ab^ |
| PF | 2-pentylfuran | 137173±9869^a^ | 117194±11761^a^ | 108489±1974^a^ | 127524±38125^a^ | 168202±34876^a^ | 143795±19054^a^ | 105345±52547^a^ | 116865±20735^a^ | 102848±12784^a^ | 192868±81253^a^ | 163715±25033^a^ | 129336±26912^a^ |
|  | Sulfur compounds |  |  |  |  |  |  |  |  |  |  |  |  |
| DMDS | dimethyl disulfide | 338±76^a^ | 424±163^a^ | 373±76^a^ | 430±234^a^ | 272±38^a^ | 318±155^a^ | 242±123^a^ | 636±240^a^ | 521±140^a^ | 436±79^a^ | 580±132^a^ | 402±101^a^ |
|  | Acids |  |  |  |  |  |  |  |  |  |  |  |  |
| BA | butanoic acid | 1490±237^cd^ | 1373±304^cd^ | 1620±540^bcd^ | 1102±499^d^ | 1095±257^d^ | 1348±97^cd^ | 3116±1047^a^ | 4668±174^a^ | 4965±2542^a^ | 3995±823^abc^ | 3830±576^abcd^ | 4423±1280^ab^ |
| 3-MBA | 3-methylbutanoic acid | 1045±148^c^ | 966±238^c^ | 1110±390^bc^ | 767±291^c^ | 792±171^c^ | 941±40^c^ | 1493±806^abc^ | 2759±326^a^ | 2609±1246^ab^ | 1924±378^abc^ | 2109±465^abc^ | 2239±569^abc^ |
| 2-MBA | 2-methylbutanoic acid | 294±37^ab^ | 263±52^b^ | 295±100^ab^ | 208±89^b^ | 216±46^b^ | 238±32^b^ | 305±182^ab^ | 580±35^ab^ | 655±300^a^ | 464±91^ab^ | 471±119^ab^ | 503±147^ab^ |
| PA | pentanoic acid | 3065±515^ab^ | 2706±681^ab^ | 3347±1451^ab^ | 2134±1010^b^ | 2247±501^b^ | 2660±245^ab^ | 6104±2875^ab^ | 8912±820^a^ | 8140±4967^ab^ | 7241±1431^ab^ | 5145±3817^ab^ | 8715±2580^ab^ |
| HXA | hexanoic acid | 13288±2862^bc^ | 11707±3038^c^ | 16177±8288^bc^ | 9375±4444^c^ | 10261±1996^c^ | 11960±1734^bc^ | 34128±14815^abc^ | 49566±12221^a^ | 34624±24159^abc^ | 32655±6502^abc^ | 37641±10049^abc^ | 43389±13199^ab^ |
|  | Pyrazines |  |  |  |  |  |  |  |  |  |  |  |  |
| P1 | pyrazine | 14±0^b^ | 14±0^b^ | 10±1^b^ | 10±4^b^ | 14±4^b^ | 26±2^a^ | nd | nd | nd | nd | nd | nd |
| P2 | 2-methylpyrazine | 265±5^b^ | 251±16^b^ | 208±28^bcd^ | 192±73^bcde^ | 246±72^bc^ | 389±20^a^ | 126±31^def^ | 82±22^f^ | 85±10^ef^ | 142±6^cdef^ | 190±37^bcde^ | 219±32^bcd^ |
| P3 | 2,5(6)-dimethylpyrazine | 2245±48^ab^ | 2161±209^ab^ | 1720±242^bc^ | 1715±709^bc^ | 2108±658^ab^ | 3054±98^a^ | 885±210^cd^ | 599±163^d^ | 528±75^d^ | 993±43^cd^ | 1323±259^bcd^ | 1578±247^bc^ |
| P4 | 2-ethylpyrazine | 24±1^b^ | 24±3^b^ | 18±2^bcd^ | 17±7^bcde^ | 23±7^b^ | 34±1^a^ | 9±1^def^ | 7±1^f^ | 8±1^ef^ | 13±1^cdef^ | 15±3^bcdef^ | 20±3^bc^ |
| P5 | 2,3-dimethylpyrazine | 29±1^b^ | 28±2^bc^ | 21±3^bcd^ | 22±9^bcd^ | 28±8^bc^ | 45±2^a^ | 11±1^de^ | 8±1^e^ | 9±1^e^ | 15±0^de^ | 17±4^cde^ | 22±3^bcd^ |
| P6 | 2-ethyl-5-methylpyrazine | 51±1^ab^ | 47±6^b^ | 43±5^bc^ | 41±19^bcd^ | 51±15^ab^ | 73±5^a^ | 24±2^cde^ | 19±3^de^ | 17±2^e^ | 32±1^bcde^ | 38±7^bcde^ | 43±7^bc^ |
| P7 | 2-ethyl-6-methylpyrazine | 208±12^ab^ | 201±31^ab^ | 156±22^bc^ | 163±74^bc^ | 202±71^ab^ | 294±22^a^ | 76±12^cd^ | 62±16^cd^ | 43±25^d^ | 95±4^cd^ | 117±25^bcd^ | 143±23^bcd^ |
| P8 | trimethylpyrazine | 457±16^b^ | 445±59^bc^ | 333±42^bcde^ | 359±157^bcd^ | 474±150^b^ | 705±38^a^ | 148±29^efg^ | 121±29^fg^ | 94±14^g^ | 179±8^defg^ | 243±45^cdefg^ | 316±53^bcdef^ |
| P9 | 2-ethyl-3-methylpyrazine | 20±1^ab^ | 21±3^ab^ | 17±2^ab^ | 14±7^ab^ | 18±4^ab^ | 29±3^ab^ | 11±1^b^ | 9±2^b^ | 35±25^a^ | 14±1^ab^ | 17±3^ab^ | 19±3^ab^ |
| P10 | 2-ethenyl-6-methylpyrazine | 43±3^a^ | 38±5^ab^ | 43±6^a^ | 36±17^ab^ | 36±11^ab^ | 43±5^a^ | 14±2^c^ | 12±2^cd^ | 9±2^c^ | 19±1^bc^ | 19±3^bc^ | 21±3^bc^ |
| P11 | 3-ethyl-2,5-dimethylpyrazine | 1192±46^ab^ | 1083±154^abc^ | 960±137^bcde^ | 973±441^bcd^ | 1173±379^abc^ | 1595±140^a^ | 398±53^ef^ | 334±89^f^ | 262±50^f^ | 488±26^def^ | 613±115^cdef^ | 755±123^bcdef^ |
| P12 | 2-methyl-6-1-propenylpyrazine | 30±2^a^ | 30±5^a^ | 28±4^ab^ | 25±11^abcd^ | 26±8^abc^ | 32±4^a^ | 13±2^cde^ | 11±3^de^ | 8±3^e^ | 14±1^bcde^ | 16±3^bcde^ | 18±3^abcde^ |
| P13 | 2,3-diethyl-5(or6)-methylpyrazine | 4±0^a^ | 4±1^a^ | 4±0^a^ | 3±2^a^ | 5±1^a^ | 6±0^a^ | nd | nd | nd | nd | nd | nd |
| P14 | 2-methyl-3,5-diethylpyrazine | 24±0^ab^ | 22±4^bc^ | 20±3^bcd^ | 18±8^bcde^ | 25±8^ab^ | 34±3^a^ | 5±2^f^ | 6±1^ef^ | 5±1^f^ | 9±1^def^ | 11±2^cdef^ | 15±4^bcdef^ |
| P15 | 3-propyl-2,5-dimethylpyrazine | 48±2^ab^ | 43±7^abc^ | 44±8^abc^ | 38±17^abcd^ | 45±14^abc^ | 56±6^a^ | 17±2^de^ | 16±5^de^ | 12±3^e^ | 23±1^cde^ | 25±5^bcde^ | 28±6^bcde^ |

*The values within the same row followed by different letters are significantly different (*p <* 0.05) according to Tukey’s test.

**nd: not detected.

**References for Supplementary Materials**

Lu, G.; Yu, T.-H.; [Ho, C.-T.](http://?), *Generation of flavor compounds by the reaction of 2-deoxyglucose with selected amino acids*, J. Agric. Food Chem., 1997, 45, 1, 233-236, https://doi.org/10.1021/jf960609c.

van Loon, W.A.M.; [Linssen, J.P.H.](http://?); [Legger, A.](http://?); [Posthumus, M.A.](http://?); [Voragen, A.G.J.](http://?), *Identification and olfactometry of French fries flavour extracted at mouth conditions*, Food Chem., 2005, 90, 3, 417-425, https://doi.org/10.1016/j.foodchem.2004.05.005

Radulovic, N.; [Blagojevic, P.](http://?); [Palic, R.](http://?), *Comparative study of the leaf volatiles of Arctostaphylos uva-ursi (L.) Spreng. and Vaccinium vitis-idaea L. (Ericaceae)*, Molecules, 2010, 15, 9, 6168-6185, https://doi.org/10.3390/molecules15096168.

Methven L.; [Tsoukka M.](http://?); [Oruna-Concha M.J.](http://?); [Parker J.K.](http://?); [Mottram D.S.](http://?), *Influence of sulfur amino acids on the volatile and nonvolatile components of cooked salmon (Salmo salar)*, J. Agric. Food Chem., 2007, 55, 4, 1427-1436, https://doi.org/10.1021/jf0625611.

Risticevic, S.; [Carasek, E.](http://?); [Pawliszyn, J.](http://?), *Headspace solid-phase microextraction-gas chromatographic-time-of-flight mass spectrometric methodology for geographical origin verification of coffee*, Anal. Chim. Acta, 2008, 617, 1-2, 72-84, https://doi.org/10.1016/j.aca.2008.04.009.

Boylston, T.D.; [Viniyard, B.T.](http://?), *Isolation of volatile flavor compounds from peanut butter using purge-and-trap technique* in Instrumental Methods in Food and Beverage Analysis, D. Wetzel and G. Charalambous, ed(s)., 1998, 225-243.

Elmore, J.S.; [Mottram, D.S.](http://?); [Hierro, E.](http://?), *Two-fibre solid-phase microextraction combined with gas chromatography-mass spectrometry for the analysis of volatile aroma compounds in cooked pork*, J. Chromatogr. A, 2000, 905, 1-2, 233-240, https://doi.org/10.1016/S0021-9673(00)00990-0
